# Tannins and copper sulphate as antimicrobial agents to prevent contamination of *Posidonia oceanica* seedling culture for restoration purposes

**DOI:** 10.3389/fpls.2024.1433358

**Published:** 2024-11-25

**Authors:** Adriana Alagna, Vincenzo Maximiliano Giacalone, Arturo Zenone, Marco Martinez, Giovanni D’Anna, Gaspare Buffa, Caterina Jessica Cavalca, Anna Poli, Giovanna Cristina Varese, Valeria Paola Prigione, Fabio Badalamenti

**Affiliations:** ^1^ Department of Integrative Marine Ecology, Stazione Zoologica Anton Dohrn, Palermo, Italy; ^2^ National Biodiversity Future Centre (NBFC), Palermo, Italy; ^3^ Institute for the Anthropic impacts and Sustainability in Marine Environment, IAS-CNR, Capo Granitola, Italy; ^4^ Institute for the Anthropic Impacts and Sustainability in Marine Environment, IAS-CNR, Palermo, Italy; ^5^ Institute for the Anthropic Impacts and Sustainability in Marine Environment, IAS-CNR, Castellammare del Golfo, Italy; ^6^ Department of Life Sciences and Systems Biology, University of Torino, Mycotheca Universitatis Taurinensis (MUT), Torino, Italy

**Keywords:** *Posidonia oceanica*, seed cultivation, tannins, copper sulphate, seagrass propagation, pathogen, peronosporales, *Halophytophthora*

## Abstract

Seed-based restoration methods are increasingly recognized as a relevant tool contributing to halt and reverse the loss of seagrass meadows while providing genetic and evolutionary benefit for the conservation of these habitats. *Ad-hoc* protocols aimed at maximizing the survival of plantlets obtained from seeds in cultivation systems are therefore required. Previous trials of seedling culture of *Posidonia oceanica*, the dominant seagrass of the Mediterranean Sea, recorded up to 40% loss due to mould development. In this study we aim to (i) identify the putative causal agents of seed decay and (ii) test the efficacy of copper sulphate (0.2 and 2 ppm) and of tannin-based products derived from chestnut, tara and quebracho in reducing seed and seedling decay, while assessing possible phytotoxic effects on plant development. *Halophytophthora lusitanica, H. thermoambigua* and a putative new *Halophytophtora* species were identified as possible causal agents of seed loss. The antimicrobial agents (copper and tannins) reduced seed contamination by 20%, although copper sulphate at 2 ppm strongly inhibited the root growth. Among tannins, chestnut and tara reduced seeds germination by up to 75% and decreased shoot and root development, while quebracho showed a less severe phytotoxic effect. The use of copper sulphate at 0.2 ppm is therefore recommended to prevent *P. oceanica* seedling loss in culture facilities since it reduces seed contamination with no phytotoxic effects. Our results contribute to improving the seedling culture of one the key species of the Mediterranean Sea, increasing propagule availability for restoration purposes.

## Introduction

Seagrasses are foundation species thriving in shallow coastal waters across all continents except Antarctica (McKenzie et al., 2020). They provide crucial ecosystem services such as habitat and food provision, sediment stabilization, coastal protection, nutrient uptake and water filtration (Björk et al., 2008; Koch, 2001; Lilley and Unsworth, 2014; Unsworth et al., 2019). Notably they represent a key ocean carbon sink, sequestering significant amounts of atmospheric carbon dioxide, thereby contributing to climate change mitigation (Duarte et al., 2013; Fourqurean et al., 2012).

Unfortunately, seagrass meadows are declining worldwide at an alarming rate due to multiple stressors that act at global and regional scales (Dunic et al., 2021; Waycott et al., 2009). Efforts to halt and reverse this trend include global conservation and management plans as well as active restoration of degraded beds (Bayraktarov et al., 2016; Tan et al., 2020; Waltham et al., 2020).

Among restoration strategies seed-based methods are increasingly recognized for providing ecological, genetic and evolutionary benefits for seagrass conservation (Marion and Orth, 2010; Pereda-Briones et al., 2020; Statton et al., 2013; Tanner and Parham, 2010). Sexual propagules ensure the maintenance of genetic diversity of restored populations, which has been positively related to higher resistance and resilience against disturbances (Hughes and Stachowicz, 2004; Reusch et al., 2005). Moreover, higher genetic diversity enhances the recovery of structural and functional traits in restored seagrass populations (Reynolds et al., 2012a, 2012b; Williams, 2001).

Seed-based techniques include collecting and sowing seeds directly in the field, as well as processing and rearing seeds in culturing facilities to obtain seedlings, plantlets, or adult individuals for transplantation (Alagna et al., 2020; Balestri and Lardicci, 2012; van Katwijk et al., 2021). Culturing plantlets from seeds in controlled environments helps to overcome early life stage bottlenecks, increasing germination, persistence, and development (van Katwijk et al., 2016). Proper protocols and treatments aimed at maximizing survival and development in rearing systems are urgently required, including seed processing treatments like disinfection, dormancy break, and enhancement with growth promoters (Kettenring and Tarsa, 2020; Pazzaglia et al., 2022; Provera et al., 2024; Tan et al., 2020).

*Posidonia oceanica* (L.) Delile is an endemic seagrass of the Mediterranean Sea, thriving from the surface to 40 meters depth, forming submerged meadows that constitute the base for diverse and productive ecosystems in coastal areas (Boudouresque et al., 2006). Both the species and its habitat are protected under international agreements (the Barcelona and the Bern Conventions, the European Commission Habitats Directive, 92/43/EEC, the Marine Strategy Framework Directive, 2008/56/EC).

*Posidonia oceanica* produces large, buoyant fruits that act as dispersal propagules and contain a non-dormant seed, which is released to the seabed upon maturity (Guerrero-Meseguer et al., 2018). Seed dispersal, settlement, and establishment are critical phases that limit recruitment to the adult stage (Almela et al., 2008; Orth et al., 2006). The high variability of meadow flowering (Balestri, 2004; Diaz-Almela et al., 2006) and the rarity of direct observation of seedling recruitment led to the belief that the reproductive success of this clonal species was infrequent. However, evidence suggesting an increase in meadow reproductive efforts potentially driven by rising temperatures (Diaz-Almela et al., 2007) as well as an increasing number of records of successful recruitment by sexual propagules (Balestri et al., 2017; Pereda-Briones et al., 2020) lead to a reconsideration of the contribution of sexual reproduction in maintaining natural populations. At the same time interest is growing in using *P. oceanica* plantlets reared from beach-cast seeds in restoration initiatives (Escandell-Westcott et al., 2023; Terrados et al., 2013). As beach-cast seeds have very small chances of returning to the sea and recruiting on adjacent shallow soft bottoms (Alagna et al., 2015; Balestri et al., 2017; Pereda Briones et al., 2018), their collection is not expected to have a negative impact on parental stands.

Previous indoor cultivation experiences have shown up to 40% seed and seedling loss due to mould development during the early weeks after collection (Alagna pers. observation.). This study tested the effect of two potential antimicrobial agents, namely copper and tannins, on reducing microorganism infections and maximizing the survival rate of early life stages of *P. oceanica* in culture facilities. Copper sulphate has been used as fungicide in terrestrial ecosystems (Lawrence et al., 2017) and was effective in reducing oomycete pathogen infection in *Zostera marina* seeds (Govers et al., 2017). Tannins, widely present in plants, have antibacterial, antifungal and antiviral properties (Aires et al., 2011; Bhalodia and Shukla, 2011; Farha et al., 2020; Funatogawa et al., 2004) but their application in seagrass studies is unexplored.

This study aimed to i) isolate and identify the potential causal agents of seed decay; ii) test the ability of copper sulphate and of three types of tannins to inhibit the growth *in vitro* of putative pathogens; iii) evaluate the efficacy of these agents *in vivo* in reducing contamination of *P. oceanica* seeds and seedlings while also monitoring for possible phytotoxic effects. Since temperature can influence both pathogen proliferation and plant growth (Obrępalska-Stęplowska et al., 2015; Scagel et al., 2023), we applied two rearing temperatures, -20°C representative of field conditions, and a lower temperature of 15°C, to both *in vivo* disinfection treatments and the controls. We hypothesize that copper sulphate and tannins would reduce putative pathogen growth *in vitro*. In the *in vivo* tests we expect lower decay rates in seed and seedling treated with copper sulphate and tannins compared to the controls. Furthermore, we anticipate lower decay rates at 15°C compared to 20°C, although seedling development was expected to slow down at the lower temperature.

## Materials and methods

### Isolation and identification of potential pathogens

#### Sample collection and cultivation system

**Figure 1:**
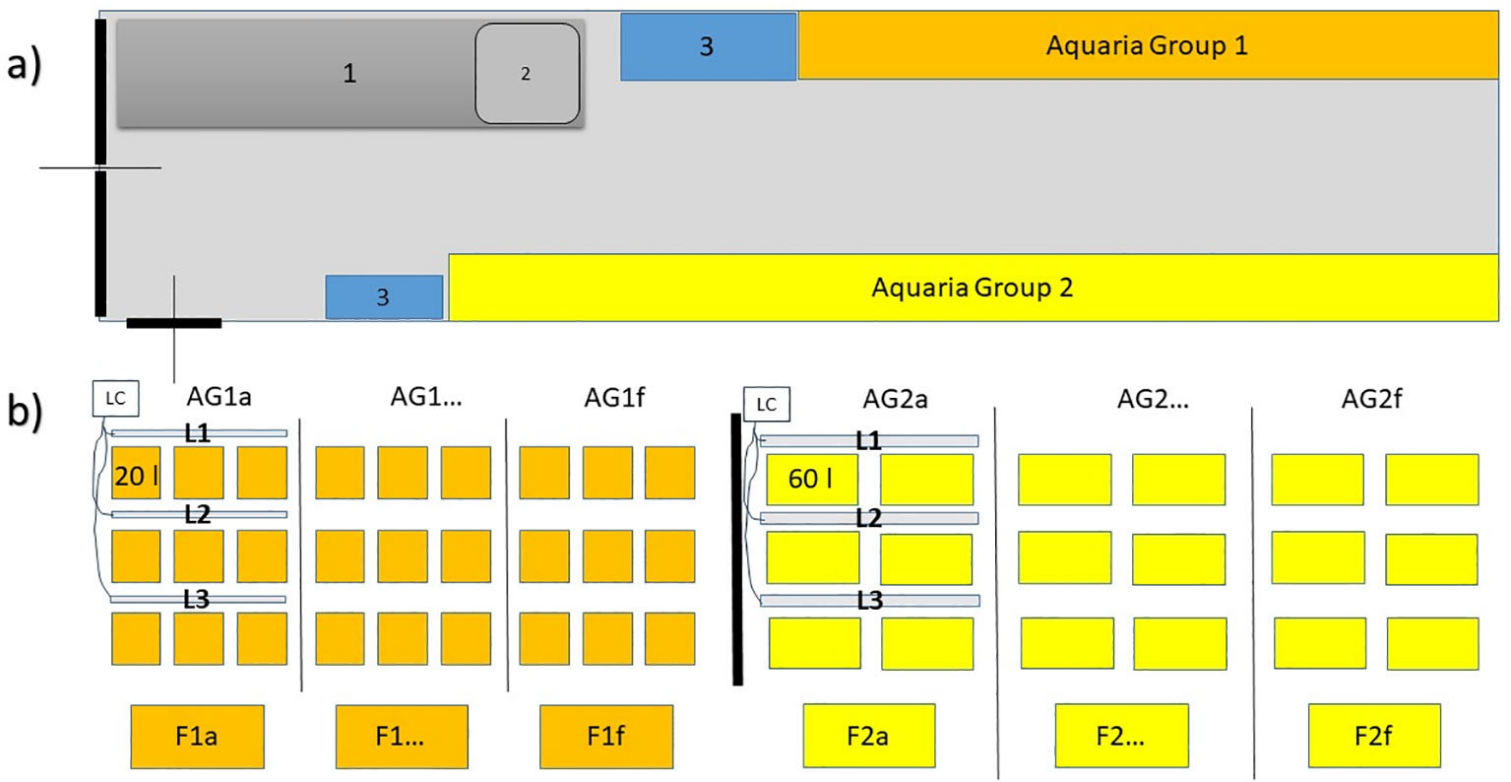
Scheme of the culture system (the content is object of patent application in Italy n. 102024000024747 filed on 05/11/2024). **(A)** Container plant with working desk (1) and washbasin (2), locker (3) and Aquaria Groups of 20 liters (AG1) and 60 liters (AG2). **(B)** details of the aquaria groups (AG1a-f; AG2a-f)7: water filtration and temperature conditioning systems (F1a-f, F2a-f), LED light bars (L1-3) and light control (LC).

Between May and July 2021, thousands of *P. oceanica* seeds were collected as drift material from sandy coasts at seven sites in north-western Sicily (Italy, from east to west: 37°59’43.31”N 13°40’30.52”E; 37°34’48.20”N 12°46’23.68”E). Seeds were extracted from fruits, if present, by cutting and opening the fruit surface, rinsed with seawater and transported in a refrigerated container (about 15°C, to avoid microorganism proliferation) to the culture facility (CNR-IAS, Capo Granitola, north-western Sicily, 37°34’23.20”N, 12°39’28.34”E). The culture system (the content is object of patent application in Italy n. 102024000024747 filed on 05/11/2024, **Figure 1**) is a 12m insulated container, inside which six groups of nine 20 l and six groups of six 60 l aquaria are housed. Each aquaria group is served by an independent water filtration and temperature conditioning system (comprising UV sterilizer, biological filter and a protein skimmer) as well as a programmable and lighting system (Eheim Led control +, “Fresh plants” with 3 led bands: white, yellow, royal blue; 9200K) in which light spectrum, intensity and photoperiod can be adjusted. Sea water is provided by a nearby calcarenitic seawater well and stocked in a tank adjacent to the container.

Following inspection, the seeds apparently in good conditions were equally distributed in the 60 L aquaria (temperature 21°C, photoperiod 12L:12D, light intensity 110 µm photons*s^-1^*m^-2^). During the first five weeks after collection seeds were checked every two days for mould infection symptoms that arecharacterized by a whitish and slimy covering causing rapid tissue rotting of seed. Infected seeds were removed from the aquaria, preserved at 4°C and sent to the *Mycotheca Universitatis Taurinensis* (MUT), Department of Life Sciences and Systems Biology, University of Torino (Italy) for mould isolation and identification.

#### Mould isolation

Sixty-three seeds were cut in four specimens in axenic condition. Two specimens were placed onto Petri dishes containing the medium CMA-P (17 g Corn Meal Agar, 5 mg Pimaricin, 250 mg Ampicillin Na, 10 mg Rifampicin, 1 L seawater) and two onto Petri dishes containing the medium CMA-Ab (17 g Corn Meal Agar, 40 mg Gentamicin sulphate, 11 mg Tazobactam, 1 L seawater). CMA-P is selective for Peronosporales and other fungus-like organisms since pimaricin prevents the growth of most fungi (Jeffers, 1986). CMA-Ab is suitable for fungi isolation since gentamicin sulphate and tazobactam avoid bacterial growth. Plates were incubated in the dark at 15°C for two weeks, periodically inspected, and developing colonies were maintained in axenic culture for molecular and morphological identification.

#### Molecular identification of moulds

Fresh mycelium carefully scraped from plates was transferred to a 2 mL Eppendorf tube and disrupted in a MM400 tissue lyzer (Retsch GmbH, Haan, Germany). Genomic DNA was extracted following the manufacturer’s instructions of a NucleoSpin kit (Macherey Nagel GmbH, Duren, DE, USA). DNA quality and quantity were measured spectrophotometrically (Infinite 200 PRO NanoQuant; Tecan, Männedorf, Switzerland), and DNA samples were stored at -20°C.

Molecular identification was performed by amplifying and sequencing the internal transcribed spacer (nrITS) and the large ribosomal subunit (nrLSU) using primer pairs ITS1/ITS4 and LR0R/LR7, respectively (White et al., 1990). PCR products were purified and sequenced at Macrogen Europe Laboratory (Madrid, Spain). The resulting ABI chromatograms were visually inspected, trimmed and assembled to obtain consensus sequences using Sequencer 5.0 (GeneCodes Corporation, Ann Arbor, MI, United States). The newly generated sequences were compared by BLASTn analyses (default settings) to those available in public nucleotide databases provided by the NCBI (Bethesda MD, United States). Similarity values equal or higher than 98% (e-value > e^-100^) were considered credible. Since no clear identification was reached with the two markers, a thorough phylogenetic analysis was performed as follows.

#### Phylogenetic analysis

A dataset consisting of nrITS and nrLSU was assembled based on BLASTn results and on the most recent phylogenetic study focused on *Halophytophthora* (Maia et al., 2022). Reference sequences were obtained from GenBank (www.ncbi.nlm.nih.gov/genbank/). Sequences were aligned using MUSCLE (default conditions for gap openings and gap extension penalties), implemented in MEGA X (Molecular Evolutionary Genetics Analysis), visually inspected, and manually trimmed to delimit and discard ambiguously aligned regions. Alignments were concatenated into a single data matrix with Sequence-Matrix since no incongruence was observed among single-loci phylogenetic trees. The best evolutionary model under the Akaike Information Criterion (AIC) was determined with jModelTest 2. Phylogenetic inference was estimated using Maximum Likelihood (ML; RAxML v.8.1.2 under GTR + I + G evolutionary model and 1000 bootstrap replicates) and Bayesian Inference (BI; GTR + I + G, 10 million generations). Consensus trees were visualized in FigTreev. 1.4.2 (http://tree.bio.ed.ac.uk/software/figtree). Due to a topological similarity of the two resulting trees, only Bayesian analysis with BS and BYPP values was reported.

### Effectiveness of antimicrobial agents

#### *In vitro* tests

*In vitro* tests were conducted at the *Mycotheca Universitatis Taurinensis* (MUT), Department of Life Sciences and Systems Biology, University of Torino (Italy). Ten *Halophytophthora lusitanica* strains previously isolated from *P. oceanica* seeds were pre-grown on PDASW (Potato Dextrose Agar 39 g, 1L Sea Water) for two weeks at 15°C. Following, sterile wheat grains were added to the surface of actively growing colonies and incubated for further three weeks at 15°C. Colonized grains were placed each in the centre of Petri dishes (9 cm in diameter) containing PDASW, supplemented with: (i) copper sulphate - 2.00 ppm, (ii) copper sulphate - 0.2 ppm, (iii) chestnut tannin-based product - 1% v/v, (iv) quebracho tannin-based product - 1% v/v, and (v) tara tannin-based product - 1% v/v. Strains grown on PDASW served as control. Plates were incubated at 15°C, and the radial growth of the colonies was measured every 3-4 days until reaching the edge of the plates in the controls. The experiment was conducted in triplicate. The tannin-based products employed in *in vitro* and *in vivo* tests were kindly provided by Silvateam Spa.

#### *In vivo* tests

Two *in vivo* experiments were conducted at the CNR-IAS facilities at Capo Granitola: the first to assess the effect of copper sulphate and temperature on seed infection and seedling development (possible phytotoxic effect) and the second to investigate the effect of three type of tannins and temperature on the same response variables. *Posidonia oceanica* seeds were collected as beach-cast material along the coast of Carini, north-western Sicily (38°10’14.76”N, 13°9’49.62”E) in May 2022. Seeds were extracted from the fruits, rinsed with seawater and carried in a refrigerated container (15°C) to the culture facility (CNR-IAS, Capo Granitola, **Figure 1**). Copper sulphate and tannins tests were carried out using two groups of 60 L aquaria, one group set at 15°C and one at 20°C. The photoperiod was set at 12L:12D with sunrise and sunset times at 6:30 and 18:30. Light intensity was set at 110 µm photons*s^-1^*m^-2^.

##### Copper sulphate experiment

**Figure 2:**
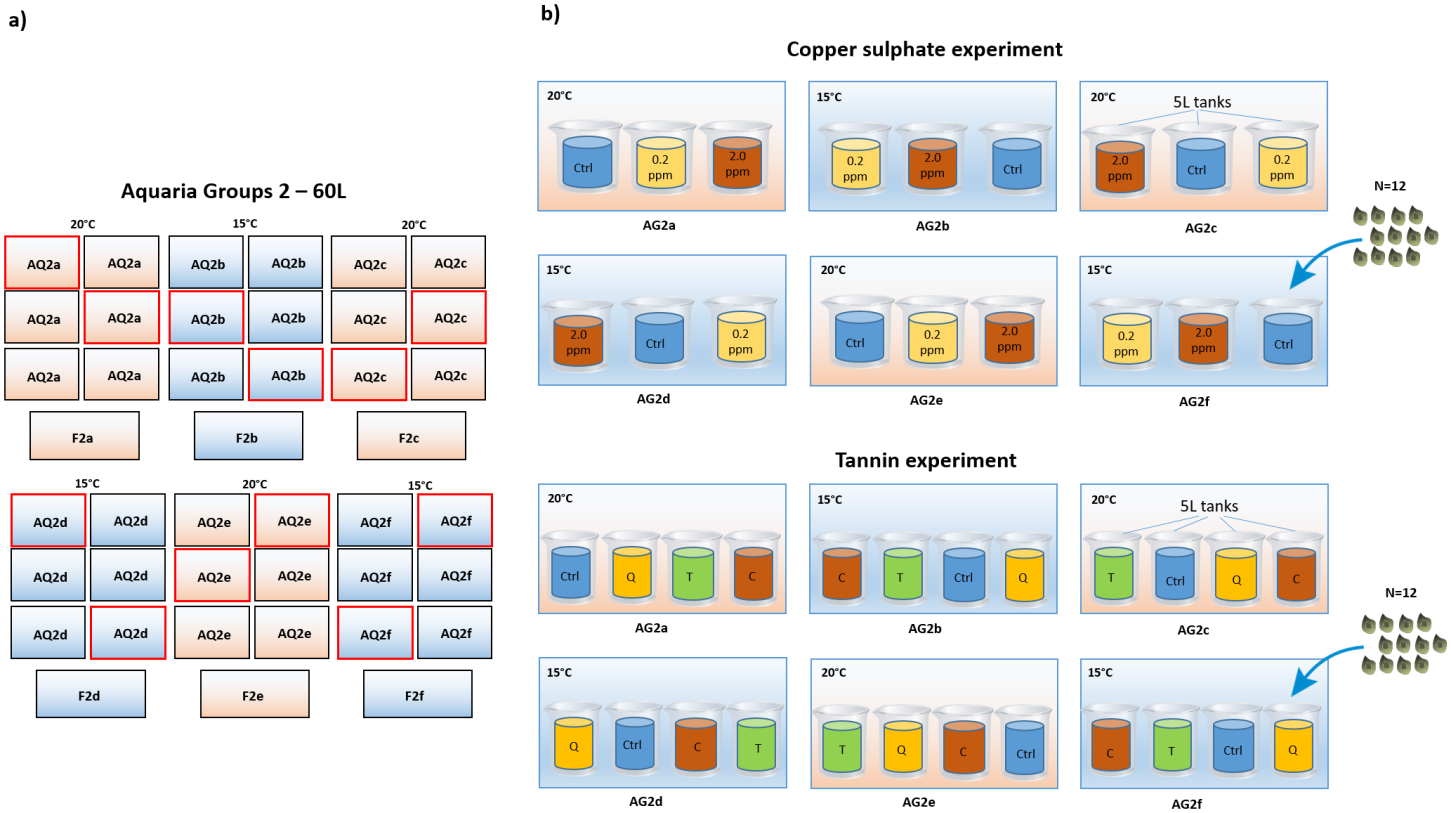
Allocation of the treatments within the culture system in *in vivo* tests. **(A)** scheme of the six groups of 60L aquaria (AQ2a-f). Aquaria used for *in vivo* tests are highlighted by a red line. **(B)** allocation of the 5L tanks to the 60L aquaria according to the disinfection treatments and the rearing temperatures (15°C or 20°C). Upper side: copper sulphate experiment, disinfection treatments: 0.2 ppm, solution of seawater and copper sulphate at 0.2 ppm concentration; 2.0 ppm, solution of seawater and copper sulphate at 2.0 ppm concentration; Ctrl, control treatment, seawater only; disinfection treatments were replicated in three 5L tanks at each rearing temperature (15°C and 20°C). Lower side: tannin experiment, disinfection treatments: C, Chestnut, solution of seawater and chestnut tannin-based product - 1% v/v; Q, Quebracho, solution of seawater and quebracho tannin-based product - 1% v/v; T, Tara, solution of seawater and tara tannin-based product - 1% v/v; Ctrl, control, seawater only; disinfection treatments were replicated in three 5L tanks at each rearing temperature (15°C and 20°C).

The effect of copper sulphate (CuSO_4_) and temperature on *P. oceanica* seed decay was investigated by rearing seeds at two concentrations (2.0, 0.2 ppm copper sulphate) and one control (0.0 ppm copper sulphate, i.e. seawater only) and two temperatures (15°C and 20°C). Disinfection and temperature were treated as orthogonal factors with 3 and 2 levels respectively, resulting in 6 different treatments, each replicated in three 5 L plastic containers (tanks). Twelve seeds of homogeneous size (seed length, measured on the longest axis of the seed) were randomly assigned to each tank. Five L independent tanks were assigned to 60 L aquaria (3 tanks for each 60 L aquaria) at 15°or 20°C according to the experimental design (a total number of 9 tanks for each rearing temperature, 3 tanks for each copper sulphate concentration, interspersed in three 60 L aquaria, **Figure 2**). Copper sulphate concentrations were based on dosages successfully applied in previous experiments to reduce pathogens incidence in the storage and maintenance of Zosteraceae seeds (Govers et al., 2017; Sullivan et al., 2022; Xu et al., 2019). Since the concentration of copper sulphate tends to decrease over time due to the adsorption of the copper ions, the copper sulphate (0.2, 2.0 ppm) and the control (0.0 ppm, seawater only) solutions were changed approximately every 3 days. The concentration of copper was checked daily with a tester (Copper checker, Hanna, HC – HI702) and adjusted if needed.

##### Tannins experiment

The effect of tannins and temperature was tested by rearing seeds at 15°C and 20°C and immersing them for 24 hours once a week in seawater (control) or in a solution of seawater and tannin-based product, 1% v/v, respectively of (i) chestnut (extracted from *Castanea sativa* wood), (ii) quebracho (extracted from *Schinopsis lorentzii* wood), and (iii) tara (extracted from *Caesalpinia spinosa* pods). These concentrations were chosen basing on previous studies showing the effectiveness of these agents in controlling pathogen infections in terrestrial plants (Miele et al., 2019). Disinfection and temperature were treated as orthogonal factors with 4 and 2 levels respectively, resulting in 8 different treatments. At the end of each exposure, seeds were transferred back to the culturing tanks with new seawater. Each treatment was replicated in three 5 L plastic containers (tanks), 12 seeds of homogeneous size were randomly assigned to each tank. Five L independent tanks were assigned to 60 L aquaria (4 tanks for each 60 L aquaria) at 15° or 20°C according to the experimental design (a total number of 12 tanks for each rearing temperature, 3 tanks for each tannin treatment plus 1 control, interspersed in six 60L aquaria, **Figure 2**).

##### Response variables

Seeds and seedlings were checked every two days and those with symptoms of mould infection were removed from the tanks. At the end of the experiments (8 weeks for the copper sulphate and 6 for the tannin experiment) the number of seedlings with symptoms was determined for each tank and summed to the number of infected seedlings removed during the experiments.

Seed germination was recorded at the end of the tannin assay, since the exposure to these agents seemed to inhibit it.

The growth performances of seedlings at the end of the two *in vivo* experiments were assessed to evaluate the effect of temperature and antimicrobial agents (possible phytotoxicity) on seedling development. Twelve individuals were randomly collected from the 3 tanks of each treatment. A total of 72 and 96 seedlings were analyzed respectively for the copper sulphate and for the tannin experiment. Seedlings were sectioned in leaves, roots and seed using a scalpel, placed on millimeter paper sheets and photographed. The images of the dissected specimens were analyzed using the software “ImageJ” (Image Processing and Analysis in Java; http://rsb.info.nih.gov/ij/). Morphological variables examined included: the number of standing leaves, maximum leaf length, maximum leaf width, total leaf area per seedling (sum of the area of all standing leaves; area of a single leaf: leaf width * leaf length) and total root length (sum of the length of the primary and the secondary roots). Seedling leaves, roots, and seed were placed in separate, numbered aluminum crucibles, and weighed. Seed, root and leaf biomasses were measured as dry weight after oven-drying at 60°C until a constant weight was reached (precision 0.0001 g). Seedling total biomass was obtained by summing seed, leaf and root biomass of each specimen. Measures were expressed in centimeters (cm), square centimeters (cm^2^) and grams (g).

### Statistical analysis

To evaluate the effect of copper sulphate and tannins on the *in vitro* growth of *Halophytophthora lusitanica* strains, we conducted a two-way analysis of variance (ANOVA), with two fixed factors, Disinfection (6 levels, namely 0.2 ppm copper sulphate, 2 ppm copper sulphate, Chestnut, Quebracho, Tara and Control) and Strain (10 levels). Pairwise comparisons were conducted via Bonferroni *post-hoc* test when significant factor effects were detected (GraphPad Prism).

For the *in vivo* experiments, exploratory data analysis revealed heteroscedasticity of variances and structuring of residuals in many of the planned analyses, necessitating the use of Generalized Linear Models (GLM) to assess the effect of disinfection treatment and rearing temperature on the response variables. The selection of the family distribution of residuals and the link function to apply in the analytical models was made based on inspection of residuals and, where applicable, AIC values selection criteria.

Poisson and quasi- Poisson distribution GLM tests with the log-link function were applied to analyze count data (i.e. the number of infected seedlings, the number of non-germinated seedlings and the number of leaves) while gamma and gaussian distributions were applied to analyze morphological and biomass variables. Given the specific distribution of the number of infected seeds, the effect of tannins was analyzed via Kruskal-Wallis test contrasting each tannin treatment with control at 15 and 20°C (Chi-squared, p-value < 0.05). The significance of the main effects and of the interaction of the explanatory variables was obtained using the analysis of deviance, type II test, with chi-squared likelihood ratio tests used to generate p-values (Wald Chi-square, p-value < 0.05). When significant effects were detected, pairwise comparisons of estimated marginal means were made via Tukey’s test (p < 0.05). Model validation through visual inspection of residuals vs fitted plot for homogeneity assumption and of residual histograms for normality distribution assumption was performed for each model. All statistical analyses were carried out using R 4.3.1 (R Core Team, 2024). Full analyses and boxplot of the response variables are provided as electronic **Supplementary Material (ES)**.

## Results

### Isolation and identification of potential pathogens (*Halophytophthora* spp.)

**Figure 3:**
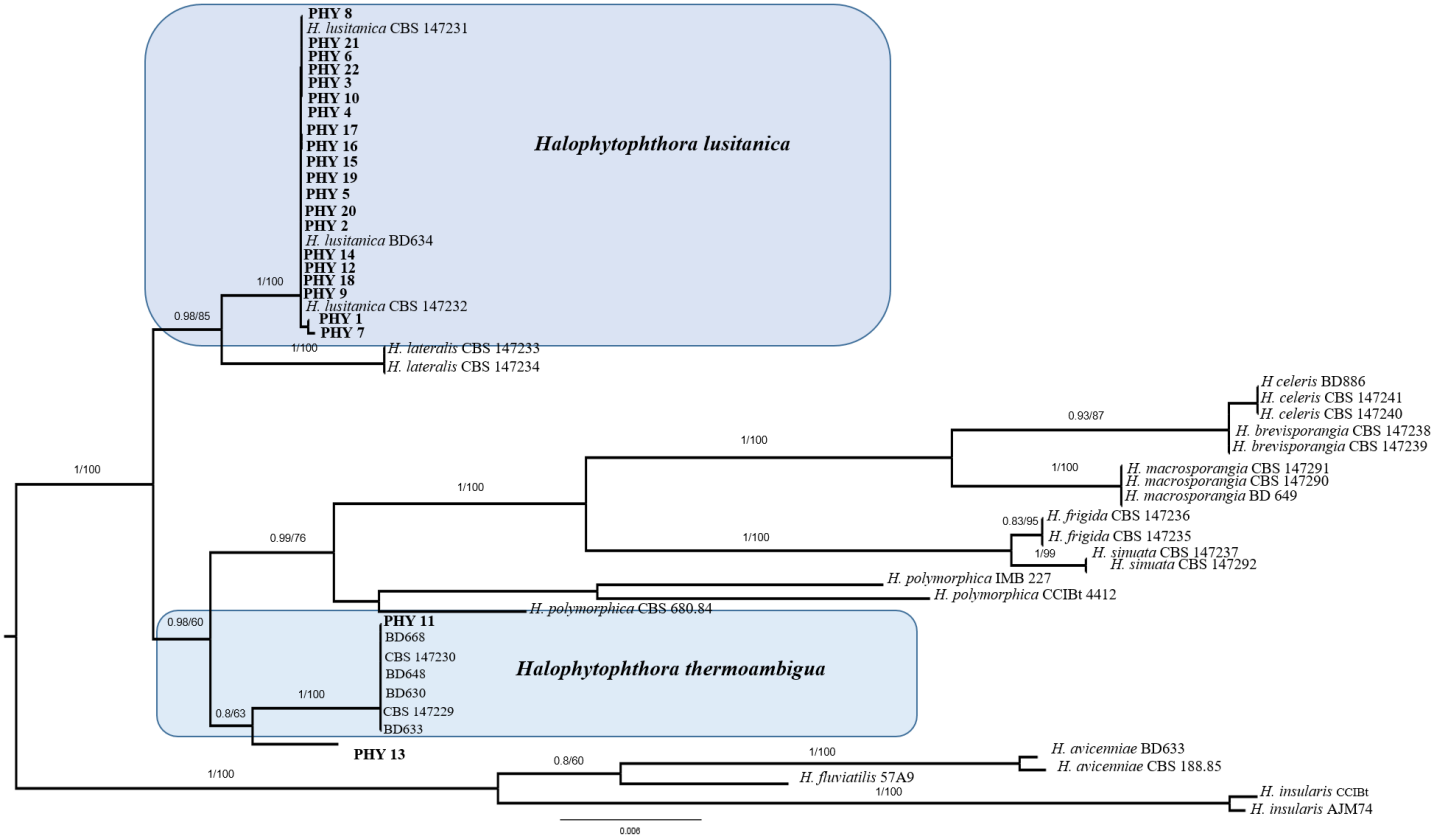
Bayesian phylogram of *Halophytophthora* sp. based on a combined nrITS, and nrLSU dataset. The tree is mid-rooted. Branch numbers indicate BYPP/BS values; Bar=expected changes per site (0.006).

Overall, 22 seeds (35%) developed colonies morphologically compatible with species of Peronosporales, 15 (24%) were colonized by filamentous fungi and/or yeasts, while both groups of microorganisms were isolated from 2 samples (3%). The fungal isolates developed on CMA-Ab belonged to genera that are widespread in marine environments, namely *Absidia, Alternaria, Aspergillus, Penicillium, Chaetomium, Cladosporium, Mucor, Trichoderma.* Being these fungi saprotrophs or secondary pathogens, no further analyses were conducted. For Peronosporales, BLASTn analyses based on nrITS and nrLSU allowed the identification only at genus level, i.e. to *Halophytophthora* sp. Phylogenetic analysis (**Figure 3**), revealed that 20 isolates clustered with *Halophytophthora lusitanica* while only one grouped with *Halophytophthora thermoambigua*. The isolate PHY13 instead was not ascribable to any known species of *Halophytophthora.*

### Effectiveness of antimicrobial agents

#### *In vitro* tests

**Figure 4:**
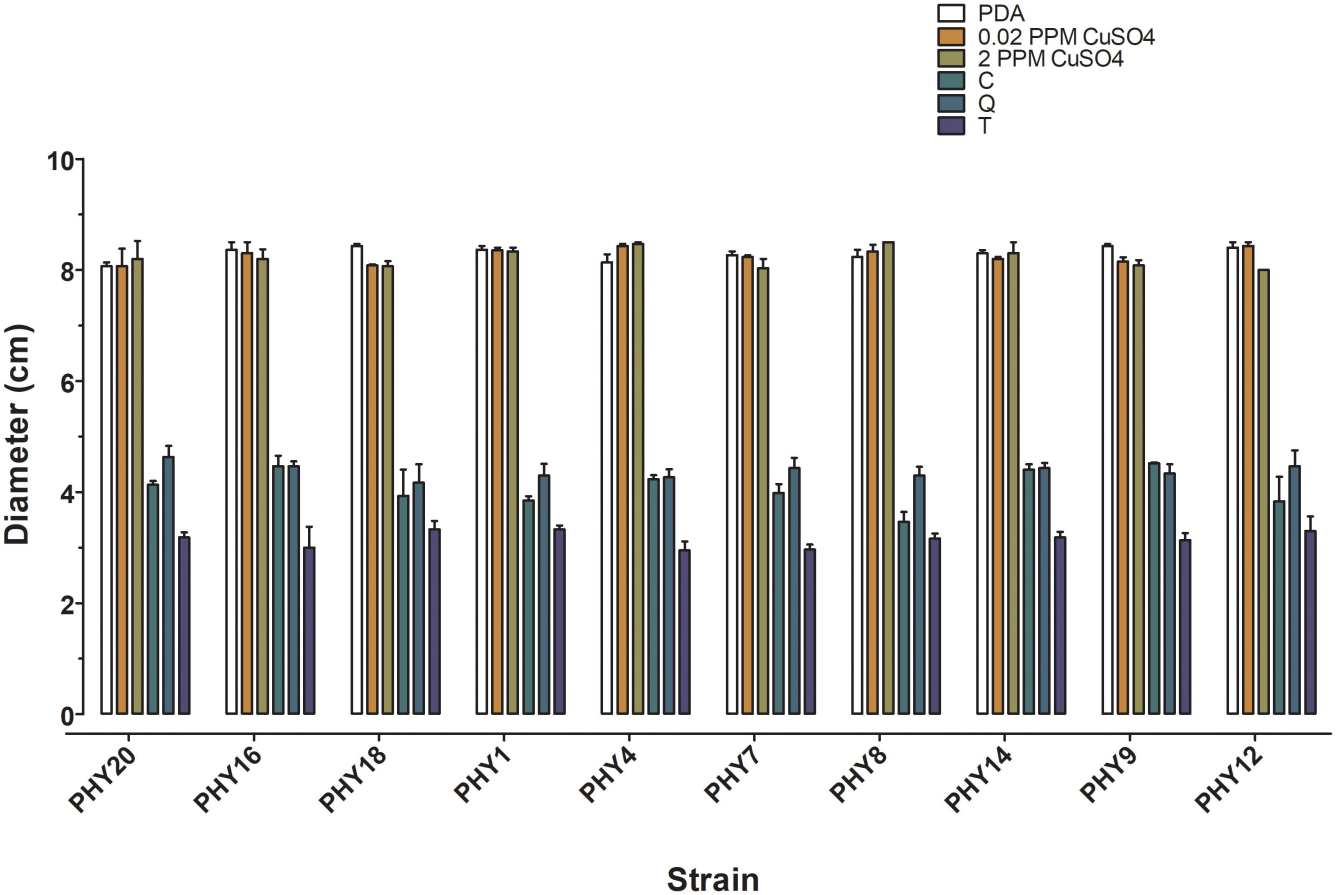
Mean values of radial growth (colony diameter in cm) of *Halophytophthora lusitanica* strains after 10 days of incubation. *PDA*, control medium; *0.2 ppm* copper sulphate, PDA + copper sulphate 0.02 ppm; *2 ppm* copper sulphate, PDA +copper sulphate 2.00 ppm; *C*, PDA + Chestnut tannin-based product, 1% v/v (C); *Q*, PDA + Quebracho tannin-based product - 1% v/v; *T*, Tara tannin-based product - 1% v/v. Bars = standard error of the mean (n=3).

Two-way analysis of variance revealed that all the strains of *H. lusitanica* displayed similar behaviour, with significant differences among disinfection treatments (p ≤ 0.01, **Figure 4**; **Supplementary Table 2 ES**). The three tannins significantly reduced colony growth by 50% compared to the control, with the Tara tannin-based product being the most effective (**Figure 4**; **Supplementary Table 2 ES**). On the contrary, the addition of copper sulphate to the culture medium at both concentrations (0.2 and 2.0 ppm) did not affect the growth of *H. lusitanica* (**Figure 4**; **Supplementary Table 2 ES**).

#### *In vivo* tests

##### Seed infection and germination

###### Effect of copper sulphate and rearing temperature

The percentage of infected seeds reached about 40% in the control treatment. Adding copper sulphate to the culture medium significantly reduced the percentage of infected seeds by 20%, with no significant differences among 0.2 and 2 ppm concentrations (**Table 1**; **Figure 5.1**; **Supplementary Table 5 ES**). Rearing seeds at 15°C significantly decreased the percentage of infected seeds by 10% with respect to 20°C (**Table 1**; **Supplementary Table 5 ES**).

**Table 1:** Summary of statistical analyses of in vivo copper sulphate and tannin disinfection experiments.

Copper sulphate experiment									
(A)									
Source of variation	df	N. of infected seedlings		N. of standing leaves		Leaf width		Length of the longest leaf	
		Chisq	p	Chisq	p	Chisq	p	Chisq	p
*Disinfection treatment (Dis)*	2	16.0221	0.0003318 ***	4.1496	0.125582	2.428	0.29701	3.078	0.21463
*Temperature (T)*	1	5.2904	0.0214429 *	10.324	0.001313 **	4.5576	0.03277 *	102.781	< 2e-16 ***
*Dis x T*	2	1.4906	0.4746016	3.3576	0.186594	2.8613	0.23915	11.404	0.00334 **
		Total leaf area		Total root length		Leaf dry weight		Root dry weight	
		Chisq	p	Chisq	p	Chisq	p	Chisq	p
*Disinfection treatment (Dis)*	2	9.882	0.007148 **	37.038	9.065e-09 ***	1.515	0.4687	20.797	3.048e-05 ***
*Temperature (T)*	1	104.203	< 2.2e-16 ***	112.322	< 2.2e-16 ***	95.844	<2e-16 ***	68.8	< 2.2e-16 ***
*Dis x T*	2	12.34	0.002091 **	17.139	0.0001898 ***	4.276	0.1179	15.064	0.0005357 ***

(B)				
Variable	Pairwise comparison			
	Between Disinfection treatments		Between Temperatures	
N. of infected seedling		Ctrl > 0.2 ppm = 2 ppm		20°C > 15°C
N. of standing leaves		–		20°C > 15°C
Leaf width		–		20°C > 15°C
Length of the longest leaf		–		20°C > 15°C
Total leaf area		–		20°C > 15°C
Total root length	Within 15°C	Ctrl = 0.2 ppm = 2 ppm	Within 0.2 ppm	20°C > 15°C
	Within 20°C	Ctrl = 0.2 ppm > 2 ppm	Within 2.0 ppm	20°C = 15°C
			Within Ctrl ppm	20°C > 15°C
Leaf dry weigth		–		20°C > 15°C
Root dry weigth	Within 15°C	Ctrl = 0.2 ppm = 2 ppm	Within 0.2 ppm	20°C > 15°C
	Within 20°C	Ctrl = 0.2 ppm > 2 ppm	Within 2.0 ppm	20°C = 15°C
			Within Ctrl ppm	20°C > 15°C

Tannin experiment									
(A)									
Source of variation	df	N. of infected seedling		N. non-germinated seeds		N. of standing leaves		Length of the longest leaf	
		Chisq	p	Chisq	p	Chisq	p	Chisq	p
Disinfection treatment (Dis)	3	19.497	**0.0002158**	88.639	<2e-16 ***	67.167	1.724e-14 ***	163.002	< 2.2e-16 ***
Temperature (T)	1	0	1	0.451	0.5019	5.64	0.0175 *	23.64	1.161e-06 ***
Dis x T	3			-1.801	1.0000	5.764	0.1236	49.179	1.195e-10 ***
Total leaf area		Total root length		Leaf dry weight		Root dry weight		Seed dry weight	
Chisq	p	Chisq	p	Chisq	p	Chisq	p	Chisq	p
113.784	< 2.2e-16 ***	186.672	< 2.2e-16 ***	119.138	< 2.2e-16 ***	154.774	< 2.2e-16 ***	3.1792	**0.36482**
20.221	6.899e-06 ***	27.76	1.373e-07 ***	14.209	0.0001636 ***	15.694	7.444e-05 ***	6.3844	0.01151 *
2.485	0.4779	66.129	2.876e-14 ***	2.431	0.487802	44.112	1.429e-09 ***	2.029	0.5664

(B)				
Variable	Pairwise comparison			
	Between Disinfection treatments		Between Temperatures	
N. of infected seedling	Within 15°C	Ctrl ≥ Quebracho ≥ Chestnut = Tara	Within Chestnut	20°C = 15°C
	Within 20°C	Ctrl > Quebracho = Chestnut = Tara	Within Ctrl	20°C > 15°C
			Within Quebracho	20°C = 15°C
			Within Tara	20°C = 15°C
N. not germinated seeds		Quebracho = Chestnut = Tara > Ctrl		–
N. of standing leaves		Ctrl > Quebracho = Chestnut = Tara		–
Length of the longest leaf	Within 15°C	Ctrl ≥ Quebracho ≥ Chestnut = Tara	Within Chestnut	20°C = 15°C
	Within 20°C	Ctrl > Quebracho = Chestnut = Tara	Within Ctrl	20°C > 15°C
			Within Quebracho	20°C = 15°C
			Within Tara	20°C = 15°C
Total leaf area		Ctrl > Quebracho > Chestnut = Tara		20°C > 15°C
Total root length	Within 15°C	Ctrl = Quebracho = Tara ≤ Chestnut	Within Chestnut	20°C = 15°C
	Within 20°C	Ctrl > Quebracho = Chestnut = Tara	Within Ctrl	20°C > 15°C
			Within Quebracho	20°C = 15°C
			Within Tara	20°C = 15°C
Leaf dry weight		Ctrl > Quebracho > Chestnut = Tara		20°C = 15°C
Root dry weight	Within 15°C	Ctrl ≥ Quebracho ≥ Chestnut = Tara	Within Chestnut	20°C = 15°C
	Within 20°C	Ctrl > Quebracho = Chestnut = Tara	Within Ctrl	20°C > 15°C
			Within Quebracho	20°C = 15°C
			Within Tara	20°C = 15°C
Seed dry weight		–		20°C > 15°C

Panel A, Chi square and P values are shown only for variables where a significant effect of the disinfection treatment, temperature, or their interaction was detected (Full analyses are available in the electronic supplementary tables). Panel B, results of pairwise planned contrasts. When the interaction term is significant, pairwise comparisons are shown only for the interaction. Significant results are in bold.

**Figure 5:**
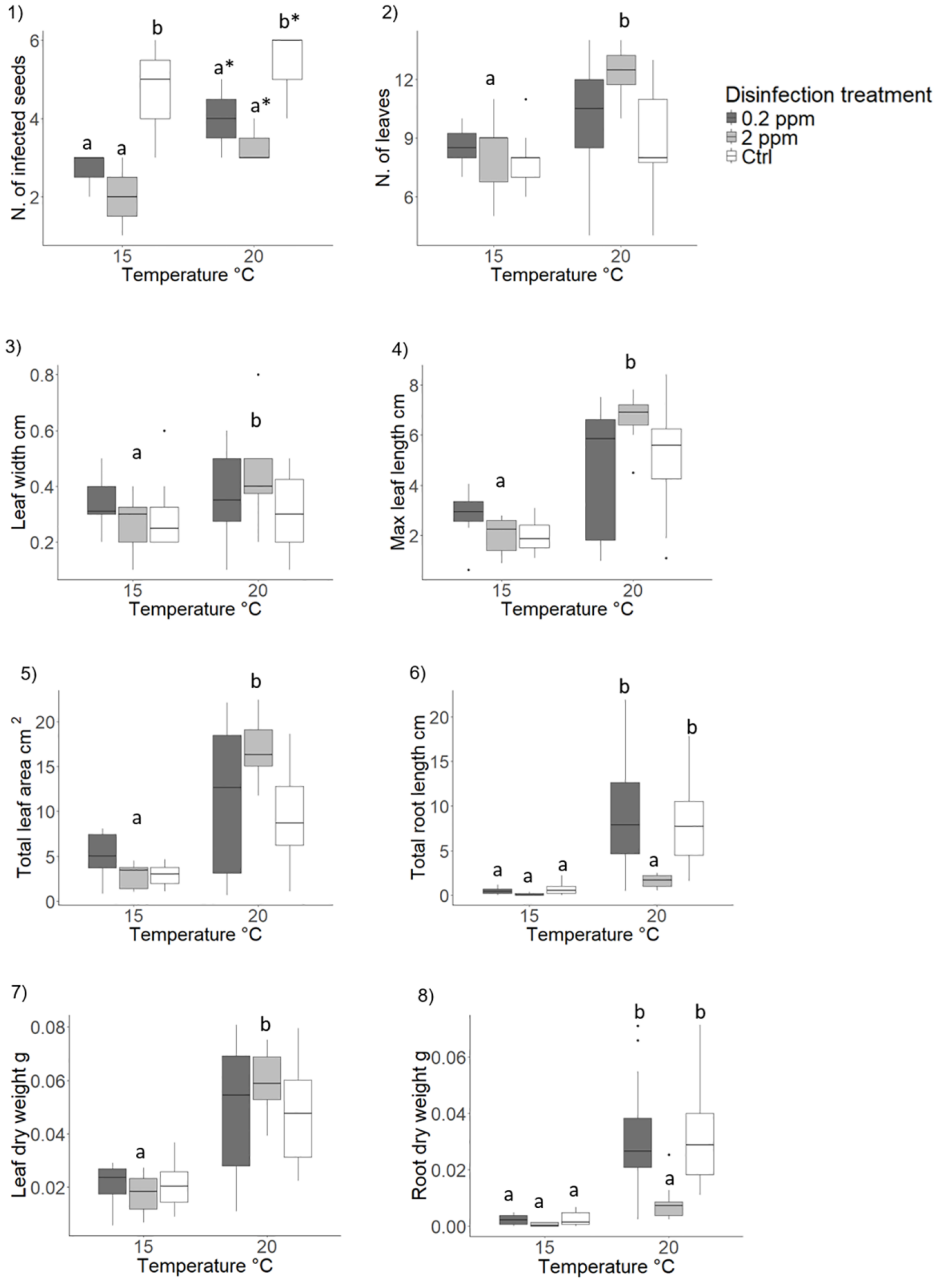
Effect of copper sulphate as antimicrobial agent and analysis of potential phytotoxic effects on *Posidonia oceanica* seedling development (morphology and biomass). Results are shown only for variables where significant effects of the disinfection treatment, the temperature or of their interaction were detected. 1), number of infected seeds; 2), number of leaves; 3), leaf width; 4), max leaf length; 5) total leaf area; 6) total root length; 7) leaf dry weight; 8), root dry weight Disinfection treatments are as follows: 0.2 ppm, solution of seawater and copper sulphate at 0.2 ppm concentration; 2.0 ppm, solution of seawater and copper sulphate at 2.0 ppm concentration; Ctrl, control treatment, seawater only. Lowercase letters and asterisks identify significant differences between means.

###### Effect of tannins and rearing temperature

At the end of the tannin experiment the percentage of infected seed in seawater only was slightly higher than 20%. Soaking seeds in solution of seawater and tannins significantly reduced the percentage of infected seeds by 20% (**Table 1**; **Figure 6.1**; **Supplementary Table 6 ES**). No infected seeds were detected in the tara and chestnut treatments (**Supplementary Table 6 ES**). A significant effect of temperature was recorded only in the controls, where the number of infected seeds was higher at 20°C compared to 15°C (**Table 1**; **Figure 6.1**; **Supplementary Table 6 ES**). Tannins also had a substantial impact on seed germination (**Table 1**; **Figure 6.2**). All seeds germinated in the controls while the percentage of not germinated seeds reached values up to 75% in the chestnut and tara treatments. In the quebracho treatment, the percentage of non-germinated seeds was lower (45%) compared to the other tannin treatments, although not statistically different, (**Table 1**; **Figure 6.2**; **Supplementary Tables 4**, **6 ES**).

**Figure 6:**
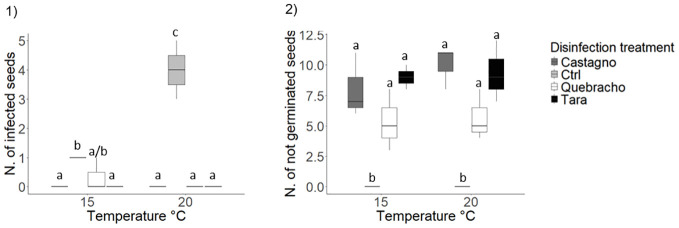
Effect s of tannins as antimicrobial agents and analysis of their effect on *Posidonia oceanica* seed germination. 1), number of infected seeds; 2), number of not germinated seeds. Disinfection treatments are as follows: Chestnut, solution of seawater and chestnut tannin-based product - 1% v/v; Quebracho, solution of seawater and quebracho tannin-based product - 1% v/v; Tara, solution of seawater and tara tannin-based product - 1% v/v; Ctrl, control, seawater only. Lowercase letters identify significant differences between means.

##### Seedling growth performances

###### Effect of copper sulphate and rearing temperature

Copper sulphate addition did not influence shoot development while temperature had a significant effect (**Table 1**; **Figure 5.2-5**; **Supplementary Tables  3, 5 ES**). Seedlings reared at 20°C developed more leaves compared to those reared at 15°C (**Table 1**; **Figure 5.2**; **Supplementary Table 5 ES**). At 20°C seedling leaves were also wider and longer resulting in a 70% increased shoot surface area compared to seedlings reared at 15°C (**Table 1**; **Figure 5.3** and **5.4**; **Supplementary Table 5 ES**).

Copper sulphate at 2.0 ppm concentration reduced root growth by 80% compared to the 0.2 ppm concentration and the control that did not differ from each other (**Table 1**; **Figure 5.6**; **Supplementary Tables 3, 5 ES**). Seedlings developed significantly longer roots at 20°C respect to 15°C in the 0.2 ppm and in the control treatment, while no difference was detected between rearing temperatures in the 2.0 ppm treatment (**Table 1**; **Figure 5.6**; **Supplementary Table 3, 5 ES**).

In accordance with morphological variables, seedlings reared at 20°C displayed a 60% higher shoot biomass compared to 15°C (**Table 1**; **Figure 5.7**; **Supplementary Table 5 ES**). At 20°C copper sulphate significantly reduced root biomass at 2.00 ppm compared to 0.2 ppm, which did not differ from the control (**Table 1**; **Figure 5.8**). No significant differences in root biomass were observed between disinfection treatments at 15°C (**Table 1**; **Figure 5.8**; **Supplementary Table 3, 5 ES**). No effects of disinfection treatment or rearing temperature were detected on seed biomass or total seedling biomass at the end of the experiment (**Supplementary Tables 2**, **3ES**; **Supplementary Figure 1 ES**).

###### Effect of tannins and rearing temperature

The number of standing leaves was reduced by more than 40% in all the tannin treatments compared to the control (**Table 1**; **Figure 7.1**; **Supplementary Table 6 ES**). Seedlings developed longer leaves in the control and in the quebracho treatment compared to chestnut and tara at 15°C (**Table 1**; **Figure 7.2**). At 20°C the control displayed longer leaves compared to all the tannin treatments which did not differ from each other (**Table 1**; **Figure 7.2**; **Supplementary Table 4,6 ES.1**). The total leaf area was greater in the control compared to the quebracho, which, in turn, was larger than in chestnut and tara treatments (**Figure 7.3**; **Supplementary Table 6 ES**). Rearing seedlings at 20°C increased leaf area by 50% with respect to 15°C (**Figure 7.3**; **Supplementary Table 6 ES**).

**Figure 7:**
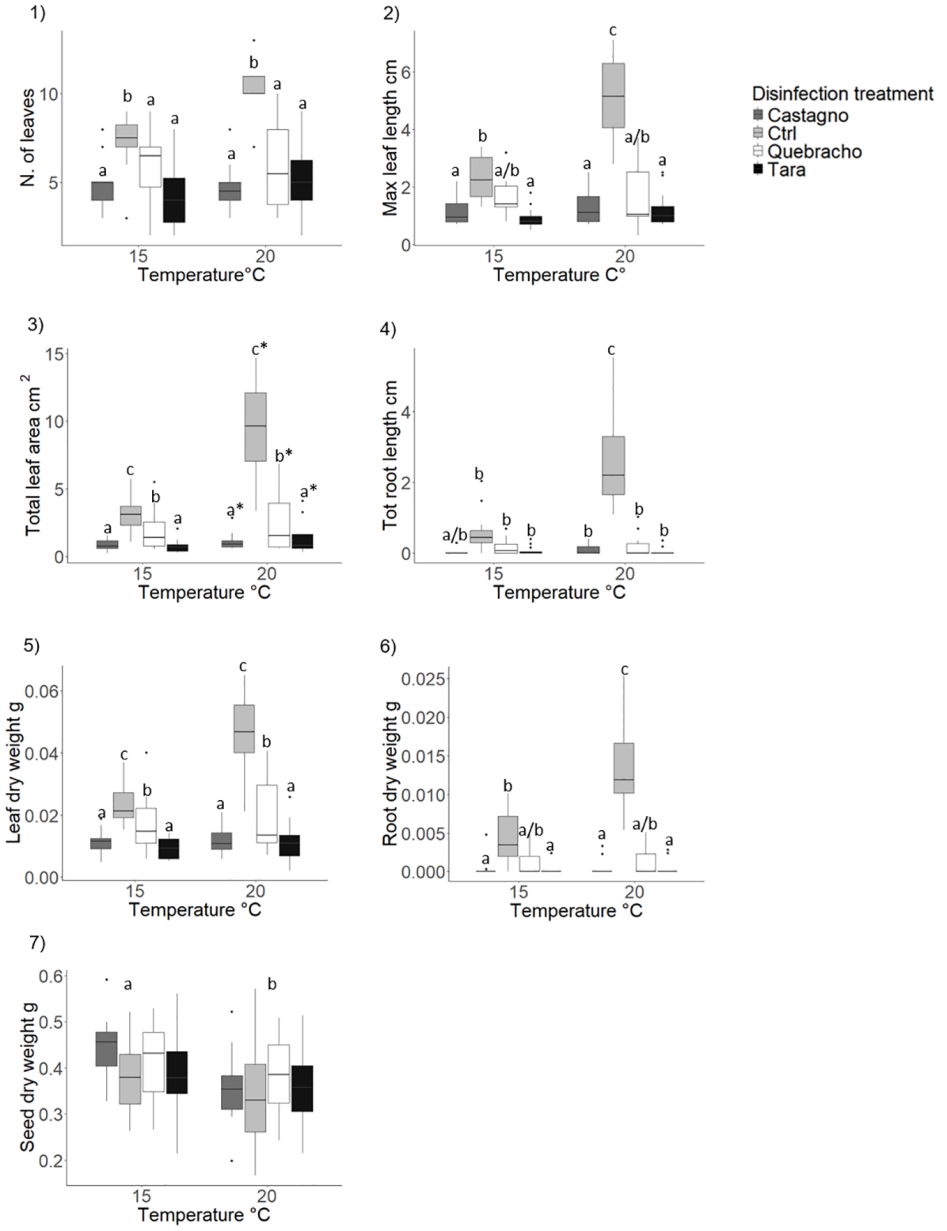
Effect of tannins on *Posidonia oceanica* seedling development (morphology and biomass). Results are shown only for variables where significant effects of the disinfection treatment, temperature or their interaction were detected. 1), number of leaves; 2), maximum leaf length; 3), total leaf area; 4), total root length; 5) leaf dry weight; 6) root dry weight; 7) seed dry weight. Disinfection treatments are as follows: Chestnut, solution of seawater and chestnut tannin-based product - 1% v/v; Quebracho, solution of seawater and quebracho tannin-based product - 1% v/v; Tara, solution of seawater and tara tannin-based product - 1% v/v; Ctrl, control, seawater only. Lowercase letters and asterisks identify significant differences between means.

At 20°C the use of all tannins strongly reduced root development compared to the control (**Table 1**; **Figure 7.4**). At 15°C only the chestnut showed a lower total root length compared to the other treatments, which did not differ from each other (**Table 1**; **Figure 7.4**; **Supplementary Table 4, 6 ES**). Higher temperature significantly increased root development only in the control (**Table 1**; **Figure 7.4**).

Shoot biomass was reduced in chestnut and tara treatments compared to quebracho, which was lower than the control (**Figure 7.5**; **Supplementary Table 6 ES**). Root dry weight was significantly reduced by all tannins with no difference across type at 20°C (**Table 1**; **Figure 7.6**). At 15°C, the quebracho treatment showed higher root biomass compared to chestnut and tara treatments and did not differ from the control (**Table 1**; **Figure 7.6**; **Supplementary Table 4, 6 ES**). A significant temperature effect was recorded only in the control, with higher root biomass at 20°C compared to 15°C (**Table 1**; **Figure 7.6**; **Supplementary Table 6 ES**). Seed biomass displayed higher values at 15°C compared to 20°C across all disinfection treatments (**Table 1**; **Figure 7.7**; **Supplementary Table 4, 6 ES**). No difference in total seedling biomass was detected at the end of the experiment (**Supplementary Table 4,6 ES**; **Supplementary Figure 2 ES**).

## Discussion and conclusions

Pathogen infection is a crucial factor affecting early seed and seedling survival in culturing systems (Govers et al., 2017, 2016; Sullivan et al., 2022; Xu et al., 2019). In this study, we isolated three species of *Halphytophthora* from *P. oceanica* for the first time: the recently described *H. lusitanica* and *H. thermoambigua* (Maia et al., 2022) and a putative novel species of the same genus. Noteworthy, these *Halophytophthora* species have never been recorded in the Mediterranean Sea. All the isolated species belong to the order Peronosporales, fungus-like organisms pathogenic to many plants and responsible for their massive die off, causing high economical damage to agriculture and the loss of complex ecosystems (Rizzo et al., 2002). Within this order, *Halophytophthora* species are mostly found in saltwater habitats (e.g. mangrove ecosystems) where they act as decomposers (Govers et al., 2016). Recently, *Halophytophthora* species have been recognised as pathogens of the seagrass *Zostera marina* across the Mediterranean Sea and the northern Atlantic (Govers et al., 2016; Man in ‘t Veld et al., 2019). In detail, Govers et al. (2016) isolated *Halphytophthora lateralis* from *Z. marina*, indicating its potential involvement in the seagrass decline by decreasing seed germination. As hypothesized for *Z. marina*, the three species of *Halophytophthora* isolated in this work could presumably be causal agents of *P. oceanica* infection under both environmental and laboratory conditions. This is the reason why we tested the effectiveness of two types of generic antimicrobial agents (copper sulphate and tannin-based products) in controlling *Halophytophthora* growth *in vitro*, with the aim of reducing the loss of vital *P. oceanica* seeds. Indeed, copper compounds are known to be lethal to zoospore, sporangia and chlamydospores of Perosporales and copper sulphate has been successfully employed to reduce *Phytophthora* and *Halophytophthora* spp. infection in *Z. marina and Heterozostera nigricaulis* seeds (Govers et al., 2017; Sullivan et al., 2022; Xu et al., 2019). Due to the survival difficulties of these organisms in axenic cultures (Turchetti et al., 2023), only strains of *H. lusitanica* remained viable. Therefore, *in vitro* tests were performed only with this species.

Copper sulphate had no effect on the growth of *H. lusitanica* colonies *in vitro* at both concentrations. Meadows et al. (2011) reported lethal effects of copper hydroxide and copper carbonate on *Phytophthora* spp. propagules within a few hours of treatment. These differences can be ascribed to a specie-specific response of *H. lusitanica* to copper compounds or to the lower effectiveness of copper sulphate on mycelium rather than propagules. Nevertheless, *in vivo*, copper sulphate generally reduced the decay of *P. oceanica* seeds at both 0.2 and 2 ppm. However, at a concentration of 2 ppm copper sulphate strongly inhibited the development of the seedling root system. Copper toxicity in plants is known to cause lipid peroxidation and DNA damage at the molecular level and to produce iron deficiency and leaf chlorosis, even in adults (Giannousi et al., 2017; Lyngby and Brix, 1984). Recently, no phytotoxic effect was recorded on seagrass seedlings treated with copper sulphate at similar dosages to control pathogens (Govers et al., 2017; Sullivan et al., 2022; Xu et al., 2019). However, a comparable toxic effect of copper was reported by Muller et al. (2001) on *Typha latifolia*, a freshwater vascular macrophyte. *T. latifolia* seeds exposed to aqueous copper sulphate at 41 µg/L (0.04 ppm) developed shorter roots, while higher Cu concentrations (0.1-0.4 ppm) completely inhibited root development (Muller et al., 2001). The authors hypothesized that copper entered the plant primarily via the root system inhibiting cell division and elongation in this region. The development of a strong root system during the early life stages is crucial for species living in high energy habitats, like *P. oceanica*. Roots not only provide essential nutrients but also anchor the plant to the substrate, thus allowing efficient recruitment once plantlets are transplanted in the field (Badalamenti et al., 2015; Zenone et al., 2022). According to the results, a fine trade-off between the beneficial and harmful effects of copper on seagrasses exists, which warrants further investigation.

Tannins are plant secondary metabolites with documented antibacterial and antifungal activity (Bhalodia and Shukla, 2011; Farha et al., 2020). In this study chestnut, tara and quebracho extracts proved effective inhibitors of *H. lusitanica* growth *in vitro* and significantly reduced the percentage of infected seeds *in vivo*. Unfortunately, a strong phytotoxic effect was detected on germination rate (up to 75%) when seeds were treated with tannins. Additionally, tannins reduced the development of seedlings, resulting in shorter shoots and roots, fewer leaves and a lower leaf surface compared to the control. The quebracho extract displayed a less severe effect on germination reduction and on inhibition of leaf and root growth. While a few investigations (Ambrin et al., 2020; Qasim et al., 2019) have described the phytotoxic activity of plant extracts due to bioactive compounds such as tannins, others (Ribeiro et al., 2022; Santiago-Medina et al., 2018) did not observed any adverse effects. Tannins, naturally present in seagrasses, play a protective function against pathogens and herbivores (Puglisi et al., 2007; Steele and Valentine, 2012). Nevertheless, they have been only roughly characterized in marine phanerogams. The toxicity of the tannin-based products employed in this study can be explained by: i) the different molecular composition and mechanism of action compared to those produced by *P. oceanica*; ii) the concentration used; iii) the exposure time; iv) the specific life stage to which the treatment was applied.

Rearing temperature influenced seed decay due to pathogens as well as the growth of seedlings in the two *in vivo* experiments. Although higher seeds decay was observed at 20°C, seedlings displayed more developed shoot and root systems compared to those reared at 15°C. In the tannin experiment, seed dry weight was lower at 20°C than at 15°C. Since seedlings rely on the carbohydrate-rich endosperm of the seed during the first months of life (Balestri et al., 2009), a higher translocation of resources from the seed to the root and shoot organs at 20°C can be hypothesized.

In conclusion, rearing *P. oceanica* seeds in a solution of copper sulphate and seawater at low concentration (0.2 ppm) during the first weeks of life is recommended to reduce seedling loss due to pathogen infection in culture facilities. Although the use of tannins proved effective *in vitro* in inhibiting the growth of *H. lusitanica*, one of the possible pathogens involved in seed mouldering, and in reducing the percentage of seeds and seedlings decay *in vivo*, these compounds showed a strong phytotoxic effect at the tested concentration, reducing seed germination and decreasing the development of both seedling shoot and root system. Among tannin products, the quebracho extract showed the least severe phytotoxic effect. Future research will aim to evaluate whether using lower concentrations of this tannin or a shorter exposure period could be effective in controlling pathogen infections without inhibiting plant development. Furthermore, specific tests will be set up to evaluate the real pathogenicity of *H. lusitanica* towards *P. oceanica* since there is a need for further investigations on the role of *Halophytophthora* spp. and, more generally, of Peronosporales in seagrass decay.

## Data Availability

The raw data supporting the conclusions of this article will be made available by the authors, without undue reservation.
