# Supplementary material for: Tannins and copper sulphate as antimicrobial agents to prevent contamination of Posidonia oceanica seedling culture for restoration purposes
Source: Front Plant Sci. 2024 Nov 25;15:1433358. doi: 10.3389/fpls.2024.1433358 (PMC11625593; doi:10.3389/fpls.2024.1433358)
Supplement: Supplementary file 1 [file DataSheet1.docx]

**Electronic supplementary materials**

Table 1 ES. Results of the two way anova and pairwise comparisons of in vitro essays. a) main analysis, b) Bonferroni posttests. Significant results are in bold.

| a) |  |  |
| --- | --- | --- |
| **Source of Variation** | **% of total variation** | **P** |
| Interaction | 0.58 | 0.0948 |
| TREATMENT | 98.22 | **< 0.0001** |
| STRAIN | 0.05 | 0.7703 |

| b) |  |  |  |  |  |  |
| --- | --- | --- | --- | --- | --- | --- |
| **Ctrl vs 0.02 PPM CuSO4** | | |  | **0.02 PPM CuSO4 vs 2 PPM CuSO4** | | |
| **STRAIN** | **P value** | **Summary** |  | **STRAIN** | **P value** | **Summary** |
| PHY20 | P > 0.05 | ns |  | PHY20 | P > 0.05 | ns |
| PHY16 | P > 0.05 | ns |  | PHY16 | P > 0.05 | ns |
| PHY18 | P > 0.05 | ns |  | PHY18 | P > 0.05 | ns |
| PHY1 | P > 0.05 | ns |  | PHY1 | P > 0.05 | ns |
| PHY4 | P > 0.05 | ns |  | PHY4 | P > 0.05 | ns |
| PHY7 | P > 0.05 | ns |  | PHY7 | P > 0.05 | ns |
| PHY8 | P > 0.05 | ns |  | PHY8 | P > 0.05 | ns |
| PHY14 | P > 0.05 | ns |  | PHY14 | P > 0.05 | ns |
| PHY9 | P > 0.05 | ns |  | PHY9 | P > 0.05 | ns |
| PHY12 | P > 0.05 | ns |  | PHY12 | P > 0.05 | ns |
|  |  |  |  |  |  |  |
| **Ctrl vs 2 PPM CuSO4** | |  |  | **0.02 PPM CuSO4 vs C** | | |
| **STRAIN** | **P value** | **Summary** |  | **STRAIN** | **P value** | **Summary** |
| PHY20 | P > 0.05 | ns |  | PHY20 | **P<0.001** | *** |
| PHY16 | P > 0.05 | ns |  | PHY16 | **P<0.001** | *** |
| PHY18 | P > 0.05 | ns |  | PHY18 | **P<0.001** | *** |
| PHY1 | P > 0.05 | ns |  | PHY1 | **P<0.001** | *** |
| PHY4 | P > 0.05 | ns |  | PHY4 | **P<0.001** | *** |
| PHY7 | P > 0.05 | ns |  | PHY7 | **P<0.001** | *** |
| PHY8 | P > 0.05 | ns |  | PHY8 | **P<0.001** | *** |
| PHY14 | P > 0.05 | ns |  | PHY14 | **P<0.001** | *** |
| PHY9 | P > 0.05 | ns |  | PHY9 | **P<0.001** | *** |
| PHY12 | P > 0.05 | ns |  | PHY12 | **P<0.001** | *** |
|  |  |  |  |  |  |  |
| **Ctrl vs C** |  |  |  | **0.02 PPM CuSO4 vs Q** | | |
| **STRAIN** | **P value** | **Summary** |  | **STRAIN** | **P value** | **Summary** |
| PHY20 | **P<0.001** | *** |  | PHY20 | **P<0.001** | *** |
| PHY16 | **P<0.001** | *** |  | PHY16 | **P<0.001** | *** |
| PHY18 | **P<0.001** | *** |  | PHY18 | **P<0.001** | *** |
| PHY1 | **P<0.001** | *** |  | PHY1 | **P<0.001** | *** |
| PHY4 | **P<0.001** | *** |  | PHY4 | **P<0.001** | *** |
| PHY7 | **P<0.001** | *** |  | PHY7 | **P<0.001** | *** |
| PHY8 | **P<0.001** | *** |  | PHY8 | **P<0.001** | *** |
| PHY14 | **P<0.001** | *** |  | PHY14 | **P<0.001** | *** |
| PHY9 | **P<0.001** | *** |  | PHY9 | **P<0.001** | *** |
| PHY12 | **P<0.001** | *** |  | PHY12 | **P<0.001** | *** |
|  |  |  |  |  |  |  |
| **Ctrl vs Q** |  |  |  | **0.02 PPM CuSO4 vs T** | | |
| **STRAIN** | **P value** | **Summary** |  | **STRAIN** | **P value** | **Summary** |
| PHY20 | **P<0.001** | *** |  | PHY20 | **P<0.001** | *** |
| PHY16 | **P<0.001** | *** |  | PHY16 | **P<0.001** | *** |
| PHY18 | **P<0.001** | *** |  | PHY18 | **P<0.001** | *** |
| PHY1 | **P<0.001** | *** |  | PHY1 | **P<0.001** | *** |
| PHY4 | **P<0.001** | *** |  | PHY4 | **P<0.001** | *** |
| PHY7 | **P<0.001** | *** |  | PHY7 | **P<0.001** | *** |
| PHY8 | **P<0.001** | *** |  | PHY8 | **P<0.001** | *** |
| PHY14 | **P<0.001** | *** |  | PHY14 | **P<0.001** | *** |
| PHY9 | **P<0.001** | *** |  | PHY9 | **P<0.001** | *** |
| PHY12 | **P<0.001** | *** |  | PHY12 | **P<0.001** | *** |
|  |  |  |  |  |  |  |
| **Ctrl vs T** |  |  |  | **2 PPM CuSO4 vs C** | |  |
| **STRAIN** | **P value** | **Summary** |  | **STRAIN** | **P value** | **Summary** |
| PHY20 | **P<0.001** | *** |  | PHY20 | **P<0.001** | *** |
| PHY16 | **P<0.001** | *** |  | PHY16 | **P<0.001** | *** |
| PHY18 | **P<0.001** | *** |  | PHY18 | **P<0.001** | *** |
| PHY1 | **P<0.001** | *** |  | PHY1 | **P<0.001** | *** |
| PHY4 | **P<0.001** | *** |  | PHY4 | **P<0.001** | *** |
| PHY7 | **P<0.001** | *** |  | PHY7 | **P<0.001** | *** |
| PHY8 | **P<0.001** | *** |  | PHY8 | **P<0.001** | *** |
| PHY14 | **P<0.001** | *** |  | PHY14 | **P<0.001** | *** |
| PHY9 | **P<0.001** | *** |  | PHY9 | **P<0.001** | *** |
| PHY12 | **P<0.001** | *** |  | PHY12 | **P<0.001** | *** |
|  |  |  |  |  |  |  |
| **2 PPM CuSO4 vs Q** | |  |  | **2 PPM CuSO4 vs T** | |  |
| **STRAIN** | **P value** | **Summary** |  | **STRAIN** | **P value** | **Summary** |
| PHY20 | **P<0.001** | *** |  | PHY20 | **P<0.001** | *** |
| PHY16 | **P<0.001** | *** |  | PHY16 | **P<0.001** | *** |
| PHY18 | **P<0.001** | *** |  | PHY18 | **P<0.001** | *** |
| PHY1 | **P<0.001** | *** |  | PHY1 | **P<0.001** | *** |
| PHY4 | **P<0.001** | *** |  | PHY4 | **P<0.001** | *** |
| PHY7 | **P<0.001** | *** |  | PHY7 | **P<0.001** | *** |
| PHY8 | **P<0.001** | *** |  | PHY8 | **P<0.001** | *** |
| PHY14 | **P<0.001** | *** |  | PHY14 | **P<0.001** | *** |
| PHY9 | **P<0.001** | *** |  | PHY9 | **P<0.001** | *** |
| PHY12 | **P<0.001** | *** |  | PHY12 | **P<0.001** | *** |
|  |  |  |  |  |  |  |
| **C vs Q** |  |  |  | **C vs T** |  |  |
| **STRAIN** | **P value** | **Summary** |  | **STRAIN** | **P value** | **Summary** |
| PHY20 | P > 0.05 | ns |  | PHY20 | **P<0.01** | ** |
| PHY16 | P > 0.05 | ns |  | PHY16 | **P<0.001** | *** |
| PHY18 | P > 0.05 | ns |  | PHY18 | P > 0.05 | ns |
| PHY1 | P > 0.05 | ns |  | PHY1 | P > 0.05 | ns |
| PHY4 | P > 0.05 | ns |  | PHY4 | **P<0.001** | *** |
| PHY7 | P > 0.05 | ns |  | PHY7 | **P<0.001** | *** |
| PHY8 | **P<0.01** | ** |  | PHY8 | P > 0.05 | ns |
| PHY14 | P > 0.05 | ns |  | PHY14 | *P<0.001* | *** |
| PHY9 | P > 0.05 | ns |  | PHY9 | *P<0.001* | *** |
| PHY12 | P > 0.05 | ns |  | PHY12 | P > 0.05 | ns |
|  |  |  |  |  |  |  |
| **Q vs T** |  |  |  |  |  |  |
| **STRAIN** | **P value** | **Summary** |  |  |  |  |
| PHY20 | **P<0.001** | *** |  |  |  |  |
| PHY16 | **P<0.001** | *** |  |  |  |  |
| PHY18 | **P<0.01** | ** |  |  |  |  |
| PHY1 | **P<0.001** | *** |  |  |  |  |
| PHY4 | **P<0.001** | *** |  |  |  |  |
| PHY7 | **P<0.001** | *** |  |  |  |  |
| PHY8 | **P<0.001** | *** |  |  |  |  |
| PHY14 | **P<0.001** | *** |  |  |  |  |
| PHY9 | **P<0.001** | *** |  |  |  |  |
| PHY12 | **P<0.001** | *** |  |  |  |  |

Table 2 ES. Radial growth (colony diameter in cm) of *Halophytophthora lusit*anica strains after 10 days of incubation. Mean, mean value; SE, standard error; n, number of replicates. PDA, control medium; 0.2 ppm CuSO4, PDA + CuSO4 0.02 ppm; 2 ppm CuSO4, PDA + CuSO4 2.00 ppm; C, PDA + Chestnut tannin-based product, 1% v/v (C); Q, PDA + Quebracho tannin-based product - 1% v/v; T, Tara tannin-based product - 1% v/v.

|  | **PDA** | | |  | **0.02 PPM CuSO4** | | |  | **2 PPM CuSO4** | | |
| --- | --- | --- | --- | --- | --- | --- | --- | --- | --- | --- | --- |
|  | *Mean* | *SE* | *n* |  | *Mean* | *SE* | *n* |  | *Mean* | *SE* | *n* |
| PHY20 | 8,07 | 0,07 | 3 |  | 8,07 | 0,32 | 3 |  | 8,2 | 0,32 | 3 |
| PHY16 | 8,37 | 0,13 | 3 |  | 8,3 | 0,2 | 3 |  | 8,2 | 0,17 | 3 |
| PHY18 | 8,43 | 0,03 | 3 |  | 8,08 | 0,02 | 3 |  | 8,07 | 0,09 | 3 |
| PHY1 | 8,37 | 0,07 | 3 |  | 8,35 | 0,05 | 3 |  | 8,33 | 0,07 | 3 |
| PHY4 | 8,13 | 0,15 | 3 |  | 8,43 | 0,03 | 3 |  | 8,47 | 0,03 | 3 |
| PHY7 | 8,27 | 0,07 | 3 |  | 8,23 | 0,03 | 3 |  | 8,03 | 0,17 | 3 |
| PHY8 | 8,23 | 0,13 | 3 |  | 8,33 | 0,12 | 3 |  | 8,5 | 0 | 3 |
| PHY14 | 8,3 | 0,06 | 3 |  | 8,2 | 0,03 | 3 |  | 8,3 | 0,2 | 3 |
| PHY9 | 8,43 | 0,03 | 3 |  | 8,15 | 0,08 | 3 |  | 8,08 | 0,09 | 3 |
| PHY12 | 8,4 | 0,1 | 3 |  | 8,43 | 0,07 | 3 |  | 8 | 0 | 3 |
|  |  |  |  |  |  |  |  |  |  |  |  |
|  | **C** | | |  | **Q** | | |  | **T** | | |
|  | *Mean* | *SE* | *n* |  | *Mean* | *SE* | *n* |  | *Mean* | *SE* | *n* |
| PHY20 | 4,13 | 0,07 | 3 |  | 4,63 | 0,2 | 3 |  | 3,18 | 0,09 | 3 |
| PHY16 | 4,47 | 0,19 | 3 |  | 4,47 | 0,09 | 3 |  | 3 | 0,38 | 3 |
| PHY18 | 3,93 | 0,47 | 3 |  | 4,17 | 0,33 | 3 |  | 3,33 | 0,15 | 3 |
| PHY1 | 3,85 | 0,08 | 3 |  | 4,3 | 0,21 | 3 |  | 3,33 | 0,07 | 3 |
| PHY4 | 4,23 | 0,07 | 3 |  | 4,27 | 0,15 | 3 |  | 2,95 | 0,16 | 3 |
| PHY7 | 3,98 | 0,16 | 3 |  | 4,43 | 0,19 | 3 |  | 2,97 | 0,09 | 3 |
| PHY8 | 3,47 | 0,18 | 3 |  | 4,3 | 0,15 | 3 |  | 3,17 | 0,09 | 3 |
| PHY14 | 4,4 | 0,1 | 3 |  | 4,43 | 0,09 | 3 |  | 3,18 | 0,1 | 3 |
| PHY9 | 4,52 | 0,02 | 3 |  | 4,33 | 0,17 | 3 |  | 3,13 | 0,13 | 3 |
| PHY12 | 3,83 | 0,44 | 3 |  | 4,47 | 0,28 | 3 |  | 3,3 | 0,26 | 3 |

Table 3 ES. Results of the main analyses and pairwise comparisons of in vivo copper sulphate experiment. Significant results are in bold.

| **Seedling infection (GLM, analysis of deviance, type II test, Wald Chi-square test)** | | |  |
| --- | --- | --- | --- |
|  |  |  |  |
| **Source** | **df** | **Chisq** | **p** |
| Disinfection treatment (Dis) | 2 | 16,0221 | **0.0003318 ***** |
| Temperature (T) | 1 | 5,2904 | **0.0214429 *** |
| Dis x T | 2 | 1,4906 | 0,4746016 |
|  |  |  |  |
|  |  |  |  |
| **Pair-wise contrasts (Tukey's test )** | | |  |
| Term 'Dis' for pairs of levels of factor 'Disinfection treatment' | | |  |
|  | **t** | **P** |  |
| 0.2 ppm, 2 ppm | 1.272 | 0.4111 |  |
| 2 ppm, Ctrl | -3.882 | **0.0003** |  |
| 0.2 ppm, Ctrl | -2.701 | **0.0189** |  |
|  |  |  |  |
| Term 'T' for pairs of levels of factor 'Temperature' |  |  |  |
|  | **t** | **P** |  |
| 15° C, 20° C | -0.052 | 0.0121 |  |
|  |  |  |  |
| **Seedling morphology** | | |  |
|  |  |  |  |
| **Number of standing leaves (GLM, analysis of deviance, type II test, Wald Chi-square test)** | | | |
| **Source** | **df** | **Chisq** | **p** |
| Disinfection treatment (Dis) | 2 | 41.496 | 0.125582 |
| Temperature (T) | 1 | 10.324 | **0.001313 **** |
| Dis x T | 2 | 33.576 | 0.186594 |
|  |  |  |  |
|  |  |  |  |
| **Pair-wise contrasts (Tukey's test )** | | |  |
| Term 'T' for pairs of levels of factor 'Temperature' |  |  |  |
|  | **t** | **P** |  |
| 15° C, 20° C | -3.078 | 0.0021 |  |
|  |  |  |  |
|  |  |  |  |
| **Leaf width (GLM, analysis of deviance, type II test, Wald Chi-square test)** | | |  |
| **Source** | **df** | **Chisq** | **p** |
| Disinfection treatment (Dis) | 2 | 2.428 | 0.29701 |
| Temperature (T) | 1 | 45.576 | **0.03277 *** |
| Dis x T | 2 | 28.613 | 0.23915 |
|  |  |  |  |
|  |  |  |  |
| **Pair-wise contrasts (Tukey's test )** | | |  |
| Term 'T' for pairs of levels of factor 'Temperature' |  |  |  |
|  | **t** | **P** |  |
| 15° C, 20° C | 2.076 | **0.0418** |  |
|  |  |  |  |
| **Length of the longest leaf (GLM, analysis of deviance, type II test, Wald Chi-square test)** | | | |
| **Source** | **df** | **Chisq** | **p** |
| Disinfection treatment (Dis) | 2 | 3.078 | 0.21463 |
| Temperature (T) | 1 | 102.781 | **< 2e-16 ***** |
| Dis x T | 2 | 11.404 | **0.00334 **** |
|  |  |  |  |
|  |  |  |  |
| **Pair-wise contrasts (Tukey's test )** | | |  |
| Term 'DisxT' for pairs of levels of factor 'Disinfection treatment' | | |  |
| *Within level 15°C of factor 'Temperature'* | **t** | **P** |  |
| 0.2 ppm, 2 ppm | -2.373 | 0.1808 |  |
| 2 ppm, Ctrl | -0.095 | 1 |  |
| 0.2 ppm, Ctrl | -2.461 | 0.1509 |  |
|  |  |  |  |
| *Within level 20°C of factor 'Temperature'* | **t** | **P** |  |
| 0.2 ppm, 2 ppm | 2.268 | 0.222 |  |
| 2 ppm, Ctrl | 0.599 | 0.9907 |  |
| 0.2 ppm, Ctrl | -1.7 | 0.5368 |  |
| Term 'DisxT' for pairs of levels of factor 'Temperature' | | |  |
| *Within level 0.2 ppm of factor 'Disinfection treatment'* | **t** | **P** |  |
| 15°C, 20°C | 3.201 | **0.0246** |  |
|  |  |  |  |
| *Within level 2 ppm of factor 'Disinfection treatment'* | **t** | **P** |  |
| 15°C, 20°C | 6.406 | **<.0001** |  |
|  |  |  |  |
| *Within level Ctrl of factor 'Disinfection treatment'* | **t** | **P** |  |
| 15°C, 20°C | 5.515 | **<.0001** |  |
|  |  |  |  |
|  |  |  |  |
|  |  |  |  |
| **Total leaf area (GLM, analysis of deviance, type II test, Wald Chi-square test)** | | |  |
| **Source** | **df** | **Chisq** | **p** |
| Disinfection treatment (Dis) | 2 | 9.882 | **0.007148 **** |
| Temperature (T) | 1 | 104.203 | **< 2.2e-16 ***** |
| Dis x T | 2 | 12.34 | **0.002091 **** |
|  |  |  |  |
|  |  |  |  |
| **Pair-wise contrasts (Tukey's test )** | | |  |
| Term 'DisxT' for pairs of levels of factor 'Disinfection treatment' | | |  |
| *Within level 15°C of factor 'Temperature'* | **t** | **P** |  |
| 0.2 ppm, 2 ppm | -2.781 | 0.0733 |  |
| 2 ppm, Ctrl | 0.15 | 1 |  |
| 0.2 ppm, Ctrl | -2.658 | 0.098 |  |
|  |  |  |  |
| *within level 20°C of factor 'Temperature'* | **t** | **P** |  |
| 0.2 ppm, 2 ppm | 1.936 | 0.3903 |  |
| 2 ppm, Ctrl | -2.919 | 0.0521 |  |
| 0.2 ppm, Ctrl | -1.14 | 0.8626 |  |
| Term 'DisxT' for pairs of levels of factor 'Temperature' | | |  |
| *within level 0.2 ppm of factor 'Disinfection treatment'* | **t** | **P** |  |
| 15°C, 20°C | 3.523 | **0.0097** |  |
|  |  |  |  |
| *within level 2 ppm of factor 'Disinfection treatment'* | **t** | **P** |  |
| 15°C, 20°C | 5.812 | **<.0001** |  |
|  |  |  |  |
| *within level Ctrl of factor 'Disinfection treatment'* | **t** | **P** |  |
| 15°C, 20°C | 4.549 | **0.0003** |  |
|  |  |  |  |
|  |  |  |  |
| **Total root length (GLM, analysis of deviance, type II test, Wald Chi-square test)** | | |  |
| **Source** | **df** | **Chisq** | **p** |
| Disinfection treatment (Dis) | 2 | 37.038 | **9.065e-09 ***** |
| Temperature (T) | 1 | 112.322 | **< 2.2e-16 ***** |
| Dis x T | 2 | 17.139 | **0.0001898 ***** |
|  |  |  |  |
|  |  |  |  |
| **Pair-wise contrasts (Tukey's test )** | | |  |
| Term 'DisxT' for pairs of levels of factor 'Disinfection treatment' | | |  |
| *within level 15°C of factor 'Temperature'* | **t** | **P** |  |
| 0.2 ppm, 2 ppm | -0.635 | 0.9879 |  |
| 2 ppm, Ctrl | 0.921 | 0.9398 |  |
| 0.2 ppm, Ctrl | 0.285 | 0.9997 |  |
|  |  |  |  |
| *within level 20°C of factor 'Temperature'* | **t** | **P** |  |
| 0.2 ppm, 2 ppm | -6.473 | **<.0001** |  |
| 2 ppm, Ctrl | 6.213 | **<.0001** |  |
| 0.2 ppm, Ctrl | -0.264 | 0.9998 |  |
| Term 'DisxT' for pairs of levels of factor 'Temperature' | | |  |
| *within level 0.2 ppm of factor 'Disinfection treatment'* | **t** | **P** |  |
| 15°C, 20°C | 8.086 | **<.0001** |  |
|  |  |  |  |
| *within level 2 ppm of factor 'Disinfection treatment'* | **t** | **P** |  |
| 15°C, 20°C | 2.283 | 0.2158 |  |
|  |  |  |  |
| *within level Ctrl of factor 'Disinfection treatment'* | **t** | **P** |  |
| 15°C, 20°C | 7.551 | **<.0001** |  |
|  |  |  |  |
|  |  |  |  |
| **Leaf dry weight (GLM, analysis of deviance, type II test, Wald Chi-square test)** | | |  |
| **Source** | **df** | **Chisq** | **p** |
| Disinfection treatment (Dis) | 2 | 1.515 | 0.4687 |
| Temperature (T) | 1 | 95.844 | **<2e-16 ***** |
| Dis x T | 2 | 4.276 | 0.1179 |
|  |  |  |  |
|  |  |  |  |
| **Pair-wise contrasts (Tukey's test )** | | |  |
| Term 'T' for pairs of levels of factor 'Temperature' |  |  |  |
|  | **t** | **P** |  |
| 15° C, 20° C | -9.79 | **<.0001** |  |
|  |  |  |  |
|  |  |  |  |
| **Root dry weight (GLM, analysis of deviance, type II test, Wald Chi-square test)** | | |  |
| **Source** | **df** | **Chisq** | **p** |
| Disinfection treatment (Dis) | 2 | 20.797 | **3.048e-05 ***** |
| Temperature (T) | 1 | 68.8 | **< 2.2e-16 ***** |
| Dis x T | 2 | 15.064 | **0.0005357 ***** |
|  |  |  |  |
|  |  |  |  |
| **Pair-wise contrasts (Tukey's test )** | | |  |
| Term 'DisxT' for pairs of levels of factor 'Disinfection treatment' | | |  |
| *within level 15°C of factor 'Temperature'* | **t** | **P** |  |
| 0.2 ppm, 2 ppm | -0.369 | 0.9991 |  |
| 2 ppm, Ctrl | 0.463 | 0.9972 |  |
| 0.2 ppm, Ctrl | 0.094 | 1,0000 |  |
|  |  |  |  |
| *within level 20°C of factor 'Temperature'* | **t** | **P** |  |
| 0.2 ppm, 2 ppm | -5.243 | **<.0001** |  |
| 2 ppm, Ctrl | 5.092 | **<.0001** |  |
| 0.2 ppm, Ctrl | -0.15 | 1,0000 |  |
| Term 'DisxT' for pairs of levels of factor 'Temperature' | | |  |
| *within level 0.2 ppm of factor 'Disinfection treatment'* | **t** | **P** |  |
| 15°C, 20°C | 6.495 | <.0001 |  |
|  |  |  |  |
| *within level 2 ppm of factor 'Disinfection treatment'* | **t** | **P** |  |
| 15°C, 20°C | 1.621 | 0.5881 |  |
|  |  |  |  |
| *within level Ctrl of factor 'Disinfection treatment'* | **t** | **P** |  |
| 15°C, 20°C | 6.25 | <.0001 |  |
|  |  |  |  |
|  |  |  |  |
| **Seed dry weight (GLM, analysis of deviance, type II test, Wald Chi-square test)** | | |  |
| **Source** | **df** | **Chisq** | **p** |
| Disinfection treatment (Dis) | 2 | 0.99924 | 0.6068 |
| Temperature (T) | 1 | 0.04951 | 0.8239 |
| Dis x T | 2 | 0.66563 | 0.7169 |
|  |  |  |  |
|  |  |  |  |
| **Total seedling dry weight (GLM, analysis of deviance, type II test, Wald Chi-square test)** | | |  |
| **Source** | **df** | **Chisq** | **p** |
| Disinfection treatment (Dis) | 2 | 0.99924 | 0.6068 |
| Temperature (T) | 1 | 0.04951 | 0.8239 |
| Dis x T | 2 | 0.66563 | 0.7169 |

Table 4 ES. Results of the main analyses and pairwise comparisons of in vivo tannin experiment. Significant results are in bold.

| **Seedling infection (Kruskal-Wallis rank sum test)** | | |  |  |
| --- | --- | --- | --- | --- |
|  |  |  |  |  |
| **Source** | **df** | **Chisq** | **p** |  |
| Disinfection treatment (Dis) | 3 | 19.497 | **0.0002158** |  |
| Temperature (T) | 1 | 0 | 1 |  |
|  |  |  |  |  |
|  |  |  |  |  |
| Term 'Dis' for pairs of levels of factor 'Disinfection treatment' at 15°C | | |  |  |
|  | **df** | **Chisq** | **p** |  |
| Tara, Ctrl | 1 | 5 | **0.02535** |  |
| Chestnut, Ctrl | 1 | 5 | **0.02535** |  |
| Quebracio, Ctrl | 1 | 1 | 0.1138 |  |
| Tara, Chestnut | 1 | - | - |  |
| Tara, Quebracho | 1 | 1 | 0.3173 |  |
| Chestnut, Quebracho | 1 | 1 | 0.3173 |  |
|  |  |  |  |  |
| Term 'Dis' for pairs of levels of factor 'Disinfection treatment' at 20°C | | |  |  |
|  | **df** | **Chisq** | **p** |  |
| Tara, Ctrl | 1 | 435.485 | **0.0369** |  |
| Chestnut, Ctrl | 1 | 435.485 | **0.0369** |  |
| Quebracio, Ctrl | 1 | 435.485 | **0.0369** |  |
| Tara, Chestnut | 1 | - | - |  |
| Tara, Quebracho | 1 | - | - |  |
| Chestnut, Quebracho | 1 | - | - |  |
|  |  |  |  |  |
| Term 'T' for for pairs of levels of factor 'Temperature' for each level of 'Dis' |  |  |  |  |
|  | **df** | **Chisq** | **p** |  |
| Ctrl: 15°C, 20°C | 1 | 43.548 | 0.0369 |  |
| Tara: 15°C, 20°C | 1 | - | - |  |
| Chestnut: 15°C, 20°C | 1 | - | - |  |
| Quebracho: 15°C, 20°C | 1 | 1 | 0.3173 |  |
|  |  |  |  |  |
|  |  |  |  |  |
| **Seedling germination (GLM, analysis of deviance, type II test, Wald Chi-square test)** | | | |  |
|  |  |  |  |  |
| **Source** | **df** | **Chisq** | **p** |  |
| Disinfection treatment (Dis) | 3 | 88.639 | **<2e-16 ***** |  |
| Temperature (T) | 1 | 0.451 | 0.5019 |  |
| Dis x T | 3 | -1.801 | 1,0000 |  |
|  |  |  |  |  |
|  |  |  |  |  |
| **Pair-wise contrasts (Tukey's test )** |  |  |  |  |
| Term 'Dis' for pairs of levels of factor 'Disinfection treatment' | | |  |  |
|  | **t** | **P** |  |  |
| Chestnut, Ctrl | 3.965 | **0.0004** |  |  |
| Chestnut, Quebracho | 2.174 | 0.1306 |  |  |
| Chestnut, Tara | -0.125 | 0.9993 |  |  |
| Ctrl, Quebracho | -3.475 | **0.0029** |  |  |
| Ctrl, Tara | -3.99 | **0.0004** |  |  |
| Quebracho, Tara | -2.296 | 0.099 |  |  |
|  |  |  |  |  |
|  |  |  |  |  |
| **Seedling morphology** |  |  |  |  |
|  |  |  |  |  |
| **Number of standing leaves (GLM, analysis of deviance, type II test, Wald Chi-square test)** | | | |  |
| **Source** | **df** | **Chisq** | **p** |  |
| Disinfection treatment (Dis) | 3 | 67.167 | **1.724e-14 ***** |  |
| Temperature (T) | 1 | 5.64 | **0.0175 *** |  |
| Dis x T | 3 | 5.764 | 0.1236 |  |
|  |  |  |  |  |
| **Pair-wise contrasts (Tukey's test )** |  |  |  |  |
| Term 'Dis' for pairs of levels of factor 'Disinfection treatment' | | |  |  |
|  | **t** | **P** |  |  |
| Chestnut, Ctrl | -6.434 | **<.0001** |  |  |
| Chestnut, Quebracho | -1.827 | 0.2607 |  |  |
| Chestnut, Tara | 0.372 | 0.9824 |  |  |
| Ctrl, Quebracho | 4.711 | **<.0001** |  |  |
| Ctrl, Tara | 6.765 | **<.0001** |  |  |
| Quebracho, Tara | 2.194 | 0.1249 |  |  |
|  |  |  |  |  |
| **Pair-wise contrasts (Tukey's test )** |  |  |  |  |
| Term 'T' for pairs of levels of factor 'Temperature' |  |  |  |  |
|  | **t** | **P** |  |  |
| 15° C, 20° C | -1.849 | 0.0645 |  |  |
|  |  |  |  |  |
|  |  |  |  |  |
| **Leaf width (GLM, analysis of deviance, type II test, Wald Chi-square test)** | | |  |  |
| **Source** | **df** | **Chisq** | **p** |  |
| Disinfection treatment (Dis) | 3 | 12.973 | 0.72978 |  |
| Temperature (T) | 1 | 0.1385 | 0.70979 |  |
| Dis x T | 3 | 69.499 | 0.07351 |  |
|  |  |  |  |  |
|  |  |  |  |  |
| **Length of the longest leaf (GLM, analysis of deviance, type II test, Wald Chi-square test)** | | | |  |
| **Source** | **df** | **Chisq** | **p** |  |
| Disinfection treatment (Dis) | 3 | 163.002 | **< 2.2e-16 ***** |  |
| Temperature (T) | 1 | 23.64 | **1.161e-06 ***** |  |
| Dis x T | 3 | 49.179 | **1.195e-10 ***** |  |
|  |  |  |  |  |
| **Pair-wise contrasts (Tukey's test )** |  |  |  |  |
| Term 'DisxT' for pairs of levels of factor 'Disinfection treatment' | | |  |  |
| *within level 15°C of factor 'Temperature'* | **t** | **P** |  |  |
| Chestnut, Ctrl | -3.392 | **0.0223** |  |  |
| Chestnut, Quebracho | -1.367 | 0.8697 |  |  |
| Chestnut, Tara | 0.785 | 0.9935 |  |  |
| Ctrl, Quebracho | 2.025 | 0.4715 |  |  |
| Ctrl, Tara | 4.176 | **0.0017** |  |  |
| Quebracho, Tara | 2.151 | 0.3911 |  |  |
|  |  |  |  |  |
| *within level 20°C of factor 'Temperature'* | **t** | **P** |  |  |
| Chestnut, Ctrl | -11.562 | **<.0001** |  |  |
| Chestnut, Quebracho | -1.083 | 0.9587 |  |  |
| Chestnut, Tara | 0.182 | 1,0000 |  |  |
| Ctrl, Quebracho | 10.479 | **<.0001** |  |  |
| Ctrl, Tara | 11.744 | **<.0001** |  |  |
| Quebracho, Tara | 1.266 | 0.909 |  |  |
| Term 'DisxT' for pairs of levels of factor 'Temperature' | |  |  |  |
| *within level Chestnut of factor 'Disinfection treatment'* | **t** | **P** |  |  |
| 15°C, 20°C | -0.309 | 1,0000 |  |  |
|  |  |  |  |  |
| *within level Quebracho of factor 'Disinfection treatment'* | **t** | **P** |  |  |
| 15°C, 20°C | -0.025 | 1,0000 |  |  |
|  |  |  |  |  |
| *within level Tara of factor 'Disinfection treatment'* | **t** | **P** |  |  |
| 15°C, 20°C | -0.911 | 0.9842 |  |  |
|  |  |  |  |  |
| *within level Ctrl of factor 'Disinfection treatment'* | **t** | **P** |  |  |
| 15°C, 20°C | -8.479 | **<.0001** |  |  |
|  |  |  |  |  |
|  |  |  |  |  |
| **Total leaf area per seedling (GLM, analysis of deviance, type II test, Wald Chi-square test)** | | | |  |
| **Source** | **df** | **Chisq** | **p** |  |
| Disinfection treatment (Dis) | 3 | 113.784 | **< 2.2e-16 ***** |  |
| Temperature (T) | 1 | 20.221 | **6.899e-06 ***** |  |
| Dis x T | 3 | 2.485 | 0.4779 |  |
|  |  |  |  |  |
| **Pair-wise contrasts (Tukey's test )** |  |  |  |  |
| Term 'Dis' for pairs of levels of factor 'Disinfection treatment' | | |  |  |
|  | **t** | **P** |  |  |
| Chestnut, Ctrl | 5.584 | **<.0001** |  |  |
| Chestnut, Quebracho | 3.749 | **0.0018** |  |  |
| Chestnut, Tara | 0.179 | 0.9979 |  |  |
| Ctrl, Quebracho | -3.338 | **0.0067** |  |  |
| Ctrl, Tara | -5.352 | **<.0001** |  |  |
| Quebracho, Tara | -3.525 | **0.0037** |  |  |
|  |  |  |  |  |
| **Pair-wise contrasts (Tukey's test )** |  |  |  |  |
| Term 'T' for pairs of levels of factor 'Temperature' |  |  |  |  |
|  | **t** | **P** |  |  |
| 15° C, 20° C | 2.818 | **0.006** |  |  |
|  |  |  |  |  |
|  |  |  |  |  |
| **Total root length (GLM, analysis of deviance, type II test, Wald Chi-square test)** | | | |  |
| **Source** | **df** | **Chisq** | **p** |  |
| Disinfection treatment (Dis) | 3 | 186.672 | **< 2.2e-16 ***** |  |
| Temperature (T) | 1 | 27.76 | **1.373e-07 ***** |  |
| Dis x T | 3 | 66.129 | **2.876e-14 ***** |  |
|  |  |  |  |  |
| **Pair-wise contrasts (Tukey's test )** |  |  |  |  |
| Term 'DisxT' for pairs of levels of factor 'Disinfection treatment' | | |  |  |
| *within level 15°C of factor 'Temperature'* | **t** | **P** |  |  |
| Chestnut, Ctrl | 3.35 | **0.0252** |  |  |
| Chestnut, Quebracho | 0.871 | 0.9879 |  |  |
| Chestnut, Tara | 0.27 | 1,000 |  |  |
| Ctrl, Quebracho | -2.479 | 0.2184 |  |  |
| Ctrl, Tara | -3.079 | 0.0536 |  |  |
| Quebracho, Tara | -0.601 | 0.9988 |  |  |
|  |  |  |  |  |
| *within level 20°C of factor 'Temperature'* | **t** | **P** |  |  |
| Chestnut, Ctrl | 12.619 | **<.0001** |  |  |
| Chestnut, Quebracho | 0.697 | 0.9969 |  |  |
| Chestnut, Tara | -0.266 | 1 |  |  |
| Ctrl, Quebracho | -11.926 | **<.0001** |  |  |
| Ctrl, Tara | -12.883 | **<.0001** |  |  |
| Quebracho, Tara | -0.962 | 0.9785 |  |  |
| Term 'DisxT' for pairs of levels of factor 'Temperature' | |  |  |  |
| *within level Chestnut of factor 'Disinfection treatment'* | **t** | **P** |  |  |
| 15°C, 20°C | 0.389 | 0.9999 |  |  |
|  |  |  |  |  |
| *within level Quebracho of factor 'Disinfection treatment'* | **t** | **P** |  |  |
| 15°C, 20°C | 0.214 | 1,0000 |  |  |
|  |  |  |  |  |
| *within level Tara of factor 'Disinfection treatment'* | **t** | **P** |  |  |
| 15°C, 20°C | -0.147 | 1,0000 |  |  |
|  |  |  |  |  |
| *within level Ctrl of factor 'Disinfection treatment'* | **t** | **P** |  |  |
| 15°C, 20°C | 9.669 | **<.0001** |  |  |
|  |  |  |  |  |
|  |  |  |  |  |
| **Leaf dry weight (GLM, analysis of deviance, type II test, Wald Chi-square test)** | | | |  |
| **Source** | **df** | **Chisq** | **p** |  |
| Disinfection treatment (Dis) | 3 | 119.138 | **< 2.2e-16 ***** |  |
| Temperature (T) | 1 | 14.209 | **0.0001636 ***** |  |
| Dis x T | 3 | 2.431 | 0.487802 |  |
|  |  |  |  |  |
| **Pair-wise contrasts (Tukey's test )** |  |  |  |  |
| Term 'Dis' for pairs of levels of factor 'Disinfection treatment' | | |  |  |
|  | **t** | **P** |  |  |
| Chestnut, Ctrl | 6.613 | **<.0001** |  |  |
| Chestnut, Quebracho | 3.493 | **0.0041** |  |  |
| Chestnut, Tara | -0.838 | 0.836 |  |  |
| Ctrl, Quebracho | -3.981 | **0.0008** |  |  |
| Ctrl, Tara | -7.056 | **<.0001** |  |  |
| Quebracho, Tara | -4.196 | **0.0004** |  |  |
|  |  |  |  |  |
| **Pair-wise contrasts (Tukey's test )** |  |  |  |  |
| Term 'T' for pairs of levels of factor 'Temperature' |  |  |  |  |
|  | **t** | **P** |  |  |
| 15° C, 20° C | 1.96 | 0.0532 |  |  |
|  |  |  |  |  |
|  |  |  |  |  |
| **Root dry weight (GLM, analysis of deviance, type II test, Wald Chi-square test)** | | | |  |
| **Source** | **df** | **Chisq** | **p** |  |
| Disinfection treatment (Dis) | 3 | 154.774 | **< 2.2e-16 ***** |  |
| Temperature (T) | 1 | 15.694 | **7.444e-05 ***** |  |
| Dis x T | 3 | 44.112 | **1.429e-09 ***** |  |
|  |  |  |  |  |
| **Pair-wise contrasts (Tukey's test )** |  |  |  |  |
| Term 'DisxT' for pairs of levels of factor 'Disinfection treatment' | | |  |  |
| *within level 15°C of factor 'Temperature'* | **t** | **P** |  |  |
| Chestnut, Ctrl | 3.496 | **0.0163** |  |  |
| Chestnut, Quebracho | 0.74 | 0.9955 |  |  |
| Chestnut, Tara | -0.19 | 1 |  |  |
| Ctrl, Quebracho | -2.756 | 0.1201 |  |  |
| Ctrl, Tara | -3.686 | **0.009** |  |  |
| Quebracho, Tara | -0.931 | 0.9822 |  |  |
|  |  |  |  |  |
| *within level 20°C of factor 'Temperature'* | **t** | **P** |  |  |
| Chestnut, Ctrl | 11.191 | **<.0001** |  |  |
| Chestnut, Quebracho | 0.663 | 0.9977 |  |  |
| Chestnut, Tara | -0.028 | 1 |  |  |
| Ctrl, Quebracho | -10.529 | **<.0001** |  |  |
| Ctrl, Tara | -11.219 | **<.0001** |  |  |
| Quebracho, Tara | -0.691 | 0.9971 |  |  |
| Term 'DisxT' for pairs of levels of factor 'Temperature' | |  |  |  |
| *within level Chestnut of factor 'Disinfection treatment'* | **t** | **P** |  |  |
| 15°C, 20°C | 0.035 | 1 |  |  |
|  |  |  |  |  |
| *within level Quebracho of factor 'Disinfection treatment'* | **t** | **P** |  |  |
| 15°C, 20°C | -0.042 | 1 |  |  |
|  |  |  |  |  |
| *within level Tara of factor 'Disinfection treatment'* | **t** | **P** |  |  |
| 15°C, 20°C | 0.197 | 1 |  |  |
|  |  |  |  |  |
| *within level Ctrl of factor 'Disinfection treatment'* | **t** | **P** |  |  |
| 15°C, 20°C | 7.731 | **<.0001** |  |  |
|  |  |  |  |  |
|  |  |  |  |  |
| **Seed dry weight (GLM, analysis of deviance, type II test, Wald Chi-square test)** | | | |  |
| **Source** | **df** | **Chisq** | **p** |  |
| Disinfection treatment (Dis) | 3 | 31.792 | **0.36482** |  |
| Temperature (T) | 1 | 63.844 | **0.01151 *** |  |
| Dis x T | 3 | 2.029 | 0.5664 |  |
|  |  |  |  |  |
| **Pair-wise contrasts (Tukey's test )** |  |  |  |  |
| Term 'T' for pairs of levels of factor 'Temperature' |  |  |  |  |
|  | **t** | **P** |  |  |
| 15° C, 20° C | 2.527 | **0.0133** |  |  |
|  |  |  |  |  |
|  |  |  |  |  |
| **Total seedling dry weight (GLM, analysis of deviance, type II test, Wald Chi-square test)** | | | |  |
| **Source** | **df** | **Chisq** | **p** |  |
| Disinfection treatment (Dis) | 3 | 17.181 | 0.6329 |  |
| Temperature (T) | 1 | 38.582 | **0.0495 *** |  |
| Dis x T | 3 | 29.061 | 0.4063 |  |
|  |  |  |  |  |
| **Pair-wise contrasts (Tukey's test )** |  |  |  |  |
| Term 'T' for pairs of levels of factor 'Temperature' |  |  |  |  |
|  | **t** | **P** |  |  |
| 15° C, 20° C | 1.96 | 0.0532 |  |  |

Tab. 5 ES. Percentage of infected seeds (panel a), morhopogical and biomassa variables of seedlings exposed to copper sulphate (panel b). Mean, mean value; SE, standard error; n, number of replicates. Ctrl, control treatment, seawater only; CuSO4 - 0.2 ppm, solution of seawater and copper sulphate at 0.2 ppm concentration; CuSO4 – 2 ppm, solution of seawater and copper sulphate at 2.0 ppm concentration.

| a) |  |  |  |
| --- | --- | --- | --- |
| **Infected seed (%)** | | | |
|  | *Mean* | *SE* | *n* |
| Ctrl | 41,67 | 4,30 | 6 |
| CuSO4 - 0.2 ppm | 27,78 | 3,51 | 6 |
| CuSO4 - 2 ppm | 22,22 | 3,51 | 6 |
|  |  |  |  |
| 15°C | 25,93 | 4,27 | 9 |
| 20°C | 35,19 | 3,34 | 9 |

| b) |  |  |  |  |  |  |  |  |  |  |  |  |
| --- | --- | --- | --- | --- | --- | --- | --- | --- | --- | --- | --- | --- |
|  |  | **n. of leaves** | | |  | **Leaf width (cm)** | | |  | **Leaf length (cm)** | | |
| *Disinfection* | *T* | *Mean* | *SE* | *n* |  | *Mean* | *SE* | *n* |  | *Mean* | *SE* | *n* |
| Ctrl | 15°C | 7,83 | 0,37 | 12 |  | 0,29 | 0,04 | 12 |  | 1,51 | 0,24 | 12 |
|  | 20°C | 9,00 | 0,75 | 12 |  | 0,32 | 0,04 | 12 |  | 3,41 | 0,59 | 12 |
| CuSO4 - 0.2 ppm | 15°C | 8,58 | 0,31 | 12 |  | 0,35 | 0,02 | 12 |  | 2,47 | 0,32 | 12 |
|  | 20°C | 9,92 | 0,86 | 12 |  | 0,38 | 0,05 | 12 |  | 2,86 | 0,74 | 12 |
| CuSO4 - 2 ppm | 15°C | 8,00 | 0,56 | 12 |  | 0,28 | 0,03 | 12 |  | 0,92 | 0,16 | 12 |
|  | 20°C | 12,42 | 0,38 | 12 |  | 0,42 | 0,05 | 12 |  | 4,27 | 0,87 | 12 |
|  |  |  |  |  |  |  |  |  |  |  |  |  |
|  |  | **Total leaf area (cm^2^)** | | |  | **Tot. Roots Length (cm)** | | |  | **Leaf dry weight (g)** | | |
|  |  | *Mean* | *SE* | *n* |  | *Mean* | *SE* | *n* |  | *Mean* | *SE* | *n* |
| Ctrl | 15°C | 2,92 | 0,34 | 12 |  | 0,72 | 0,21 | 12 |  | 0,0201 | 0,0024 | 12 |
|  | 20°C | 9,00 | 1,48 | 12 |  | 8,30 | 1,44 | 12 |  | 0,0472 | 0,0053 | 12 |
| CuSO4 - 0.2 ppm | 15°C | 5,14 | 0,64 | 12 |  | 0,52 | 0,12 | 12 |  | 0,0218 | 0,0020 | 12 |
|  | 20°C | 11,33 | 2,34 | 12 |  | 8,98 | 1,84 | 12 |  | 0,0497 | 0,0068 | 12 |
| CuSO4 - 2 ppm | 15°C | 2,84 | 0,37 | 12 |  | 0,13 | 0,04 | 12 |  | 0,0176 | 0,0018 | 12 |
|  | 20°C | 16,89 | 0,99 | 12 |  | 1,65 | 0,20 | 12 |  | 0,0596 | 0,0034 | 12 |
|  |  |  |  |  |  |  |  |  |  |  |  |  |
|  |  | **Root dry weight (g)** | | |  | **Seed dry weight (g)** | | |  | **Seedling dry weight (g)** | | |
|  |  | *Mean* | *SE* | *n* |  | *Mean* | *SE* | *n* |  | *Mean* | *SE* | *n* |
| Ctrl | 15°C | 0,0027 | 0,0008 | 12 |  | 0,3272 | 0,0406 | 12 |  | 0,3500 | 0,0413 | 12 |
|  | 20°C | 0,0319 | 0,0051 | 12 |  | 0,2911 | 0,0396 | 12 |  | 0,3703 | 0,0395 | 12 |
| CuSO4 - 0.2 ppm | 15°C | 0,0023 | 0,0005 | 12 |  | 0,3175 | 0,0256 | 12 |  | 0,3416 | 0,0253 | 12 |
|  | 20°C | 0,0326 | 0,0059 | 12 |  | 0,3329 | 0,0329 | 12 |  | 0,4152 | 0,0254 | 12 |
| CuSO4 - 2 ppm | 15°C | 0,0006 | 0,0002 | 12 |  | 0,2910 | 0,0287 | 12 |  | 0,3092 | 0,0296 | 12 |
|  | 20°C | 0,0081 | 0,0018 | 12 |  | 0,2937 | 0,0263 | 12 |  | 0,3615 | 0,0273 | 12 |

Tab. 6 ES. Percentage of infected seeds (panel a), morhopogical and biomassa variables of seedlings exposed to tannins (panel b). Mean, mean value; SE, standard error; n, number of replicates. Chestnut, solution of seawater and chestnut tannin-based product - 1% v/v; Quebracho, solution of seawater and quebracho tannin-based product - 1% v/v; Tara, solution of seawater and tara tannin-based product - 1% v/v; Ctrl, control, seawater only.

| a) |  |  |  |  |  |  |  |
| --- | --- | --- | --- | --- | --- | --- | --- |
|  | **Infected seed (%)** | | |  | **Not germinated seed (%)** | | |
|  | Mean | SE | n |  | Mean | SE | n |
| Chestnut | 0,00 | 0,00 | 6 |  | 75,00 | 7,76 | 6 |
| Quebracho | 1,39 | 1,39 | 6 |  | 45,83 | 7,05 | 6 |
| Tara | 0,00 | 0,00 | 6 |  | 76,39 | 5,86 | 6 |
| Ctrl | 20,83 | 5,99 | 6 |  | 0,00 | 0,00 | 6 |
|  |  |  |  |  |  |  |  |
| 15°C | 2,78 | 1,06 | 12 |  | 46,53 | 8,58 | 12 |
| 20°C | 8,33 | 4,00 | 12 |  | 52,08 | 9,54 | 12 |

| b) |  |  |  |  |  |  |  |  |  |  |  |  |
| --- | --- | --- | --- | --- | --- | --- | --- | --- | --- | --- | --- | --- |
|  |  | n. of leaves | |  |  | Leaf width (cm) | |  |  | Leaf length (cm) | |  |
| *Disinfection* | *T* | Mean | SE | n |  | Mean | SE | n |  | Mean | SE | n |
| Chestnut | 15°C | 4,92 | 0,42 | 12 |  | 0,20 | 0,02 | 12 |  | 0,89 | 0,17 | 12 |
|  | 20°C | 4,75 | 0,37 | 12 |  | 0,23 | 0,02 | 12 |  | 0,78 | 0,07 | 12 |
| Ctrl | 15°C | 7,33 | 0,48 | 12 |  | 0,24 | 0,03 | 12 |  | 1,02 | 0,09 | 12 |
|  | 20°C | 10,33 | 0,40 | 12 |  | 0,22 | 0,02 | 12 |  | 0,78 | 0,08 | 12 |
| Quebracho | 15°C | 5,75 | 0,62 | 12 |  | 0,21 | 0,02 | 12 |  | 0,85 | 0,10 | 12 |
|  | 20°C | 5,83 | 0,76 | 12 |  | 0,27 | 0,03 | 12 |  | 0,83 | 0,13 | 12 |
| Tara | 15°C | 4,25 | 0,54 | 12 |  | 0,25 | 0,02 | 12 |  | 0,68 | 0,05 | 12 |
|  | 20°C | 5,08 | 0,60 | 12 |  | 0,21 | 0,02 | 12 |  | 0,71 | 0,07 | 12 |
|  |  |  |  |  |  |  |  |  |  |  |  |  |
|  |  | Total leaf area (cmq) | |  |  | Tot. Roots Length (cm) | |  |  | Leaf dry weight (g) | |  |
| *Disinfection* | *T* | Mean | SE | n |  | Mean | SE | n |  | Mean | SE | n |
| Chestnut | 15°C | 0,85 | 0,12 | 12 |  | 0,03 | 0,03 | 12 |  | 0,0114 | 0,0011 | 12 |
|  | 20°C | 1,11 | 0,19 | 12 |  | 0,09 | 0,04 | 12 |  | 0,0116 | 0,0012 | 12 |
| Ctrl | 15°C | 3,07 | 0,38 | 12 |  | 0,62 | 0,17 | 12 |  | 0,0235 | 0,0019 | 12 |
|  | 20°C | 9,43 | 1,11 | 12 |  | 2,65 | 0,38 | 12 |  | 0,0466 | 0,0035 | 12 |
| Quebracho | 15°C | 1,95 | 0,46 | 12 |  | 0,17 | 0,07 | 12 |  | 0,0170 | 0,0028 | 12 |
|  | 20°C | 2,56 | 0,63 | 12 |  | 0,21 | 0,10 | 12 |  | 0,0196 | 0,0033 | 12 |
| Tara | 15°C | 0,77 | 0,14 | 12 |  | 0,07 | 0,04 | 12 |  | 0,0094 | 0,0010 | 12 |
|  | 20°C | 1,41 | 0,38 | 12 |  | 0,05 | 0,03 | 12 |  | 0,0116 | 0,0019 | 12 |
|  |  |  |  |  |  |  |  |  |  |  |  |  |
|  |  | Root dry weight (g) | |  |  | Seed dry weight (g) | |  |  | Seedling dry weight (g) | |  |
| *Disinfection* | *T* | Mean | SE | n |  | Mean | SE | n |  | Mean | SE | n |
| Chestnut | 15°C | 0,0004 | 0,0004 | 12 |  | 0,4451 | 0,0208 | 12 |  | 0,4569 | 0,0210 | 12 |
|  | 20°C | 0,0005 | 0,0003 | 12 |  | 0,3547 | 0,0239 | 12 |  | 0,3668 | 0,0245 | 12 |
| Ctrl | 15°C | 0,0046 | 0,0010 | 12 |  | 0,3811 | 0,0226 | 12 |  | 0,4091 | 0,0220 | 12 |
|  | 20°C | 0,0137 | 0,0019 | 12 |  | 0,3427 | 0,0348 | 12 |  | 0,4030 | 0,0382 | 12 |
| Quebracho | 15°C | 0,0013 | 0,0006 | 12 |  | 0,4142 | 0,0264 | 12 |  | 0,4325 | 0,0246 | 12 |
|  | 20°C | 0,0013 | 0,0005 | 12 |  | 0,3848 | 0,0232 | 12 |  | 0,4056 | 0,0242 | 12 |
| Tara | 15°C | 0,0002 | 0,0002 | 12 |  | 0,3884 | 0,0264 | 12 |  | 0,3979 | 0,0261 | 12 |
|  | 20°C | 0,0004 | 0,0003 | 12 |  | 0,3623 | 0,0258 | 12 |  | 0,3744 | 0,0270 | 12 |


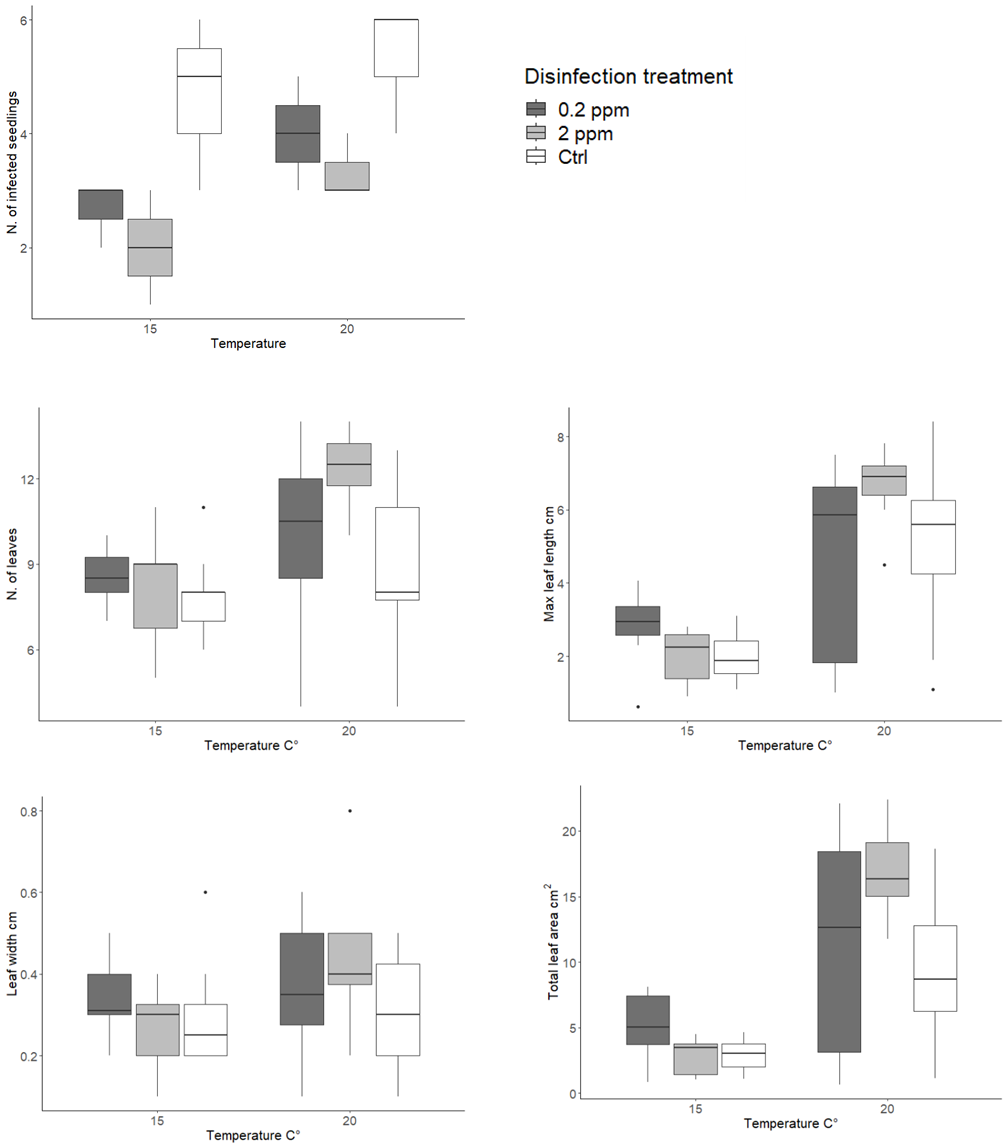


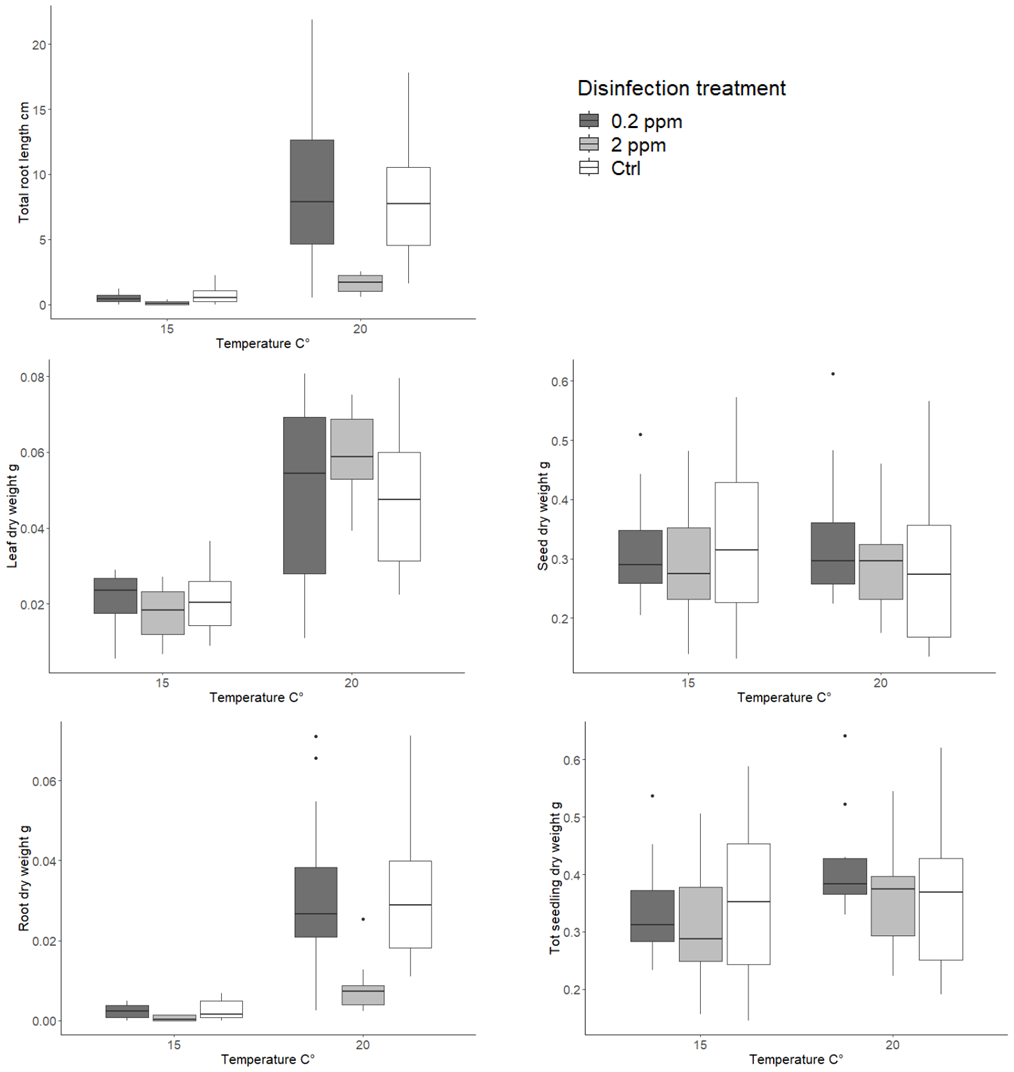


Figure ES 1. Effectiveness of copper sulphate as antimicrobial agent and analysis of possible phytotoxic effects on *Posidonia oceanica* seedling development (morphology and biomass). Disinfection treatments: 0.2 ppm, solution of seawater and copper sulphate at 0.2 ppm concentration; 2.0 ppm, solution of seawater and copper sulphate at 2.0 ppm concentration; Ctrl, control treatment, seawater only.


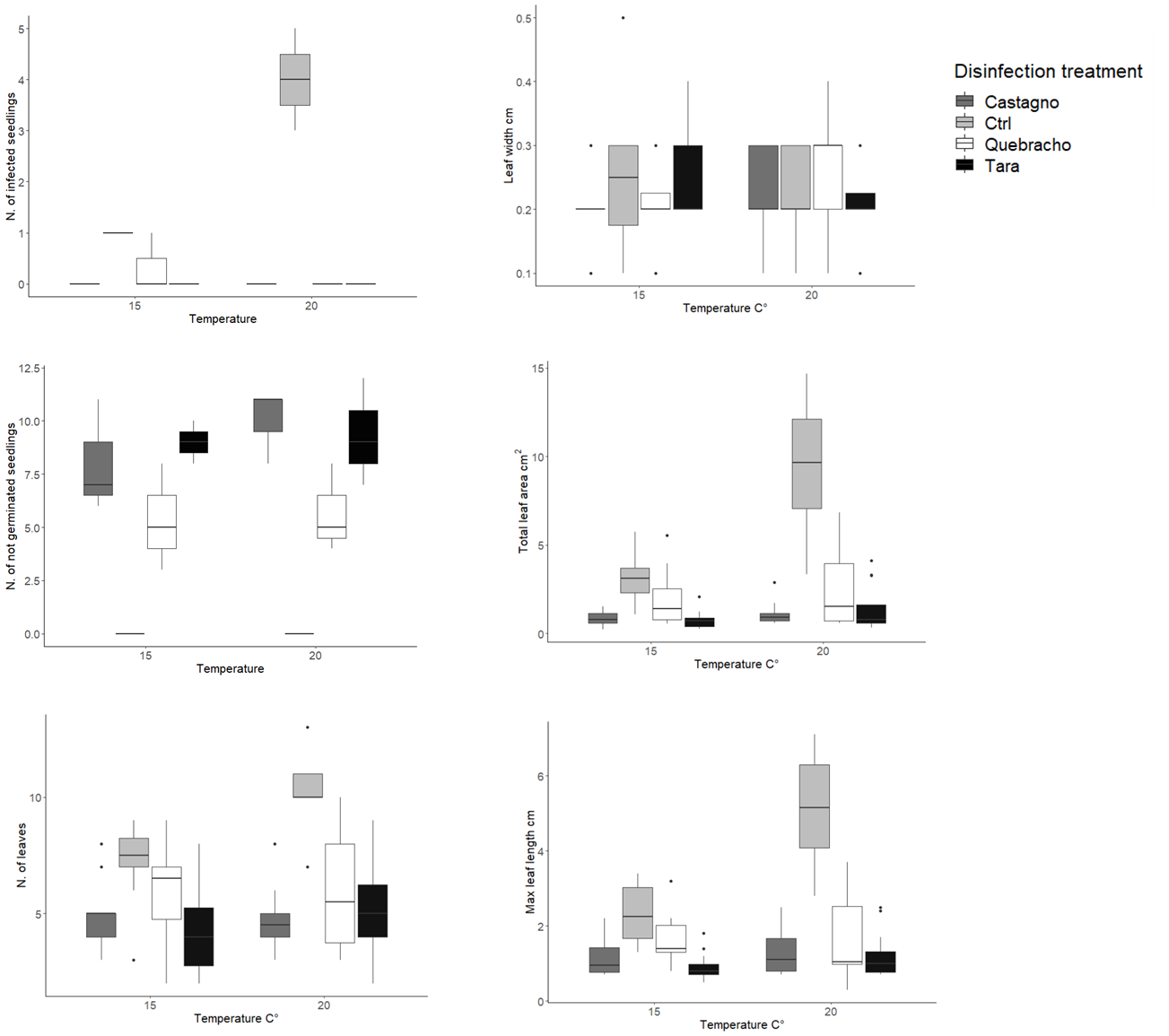


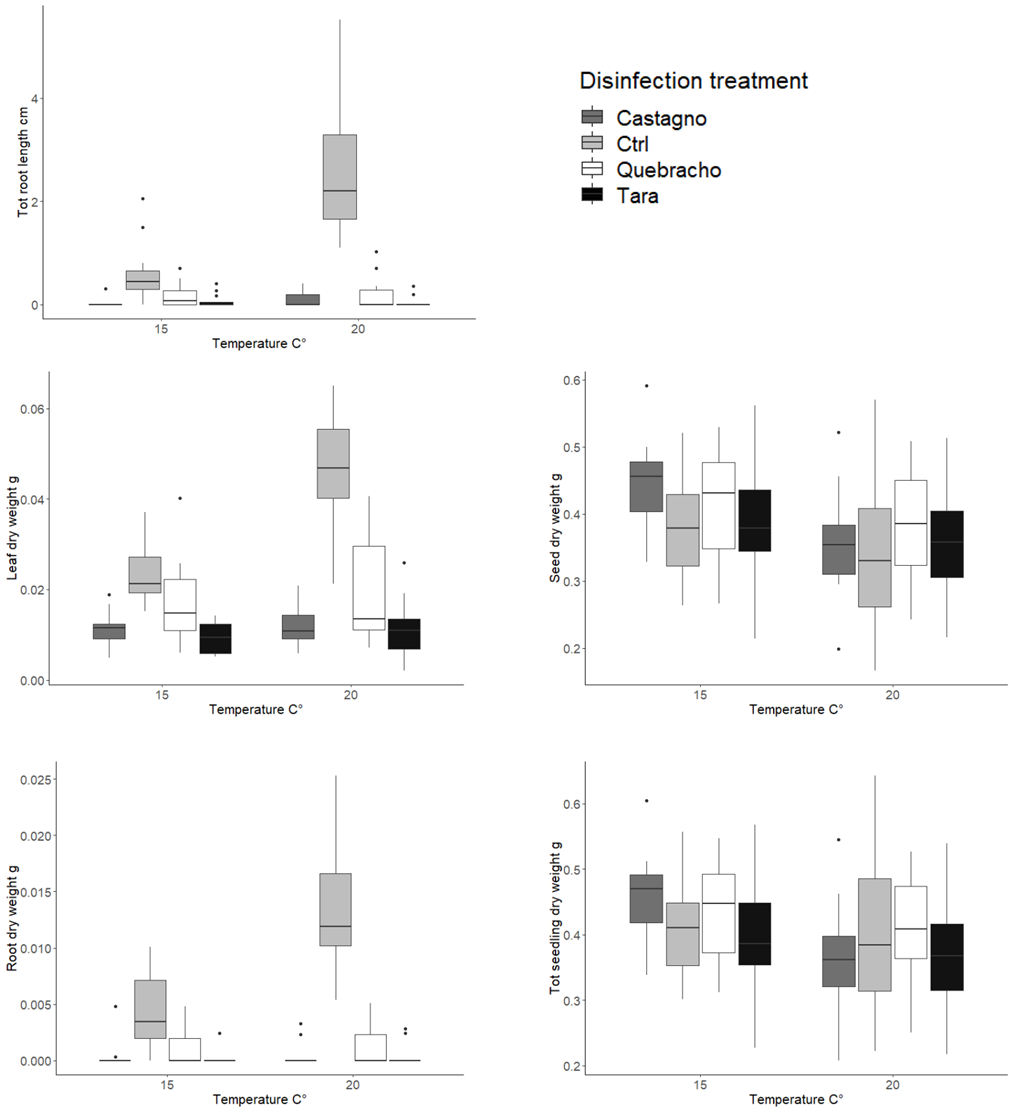


Figure 2 ES. Effectiveness of tannins as antimicrobial agents and analysis of their effect on *Posidonia oceanica* seed germination and development (morphology and biomass). Disinfection treatments: Chestnut, solution of seawater and chestnut tannin-based product (extracted from *Castanea sativa* wood) - 1% v/v; Quebracho, solution of seawater and quebracho tannin-based product (extracted from *Schinopsis lorentzii* wood) - 1% v/v ; Tara, solution of seawater and tara tannin-based product (extracted from *Caesalpinia spinosa* pods) - 1% v/v ; Ctrl, control, seawater only.
